# A dynamic *N*^*6*^-methyladenosine methylome regulates intrinsic and acquired resistance to tyrosine kinase inhibitors

**DOI:** 10.1038/s41422-018-0097-4

**Published:** 2018-10-08

**Authors:** Fei Yan, Aref Al-Kali, Zijie Zhang, Jun Liu, Jiuxia Pang, Na Zhao, Chuan He, Mark R. Litzow, Shujun Liu

**Affiliations:** 1 The Hormel Institute, University of Minnesota, Austin, MN 55912 USA; 2 Division of Hematology, Mayo Clinic, Rochester, MN 55905 USA; 3 Department of Chemistry, Department of Biochemistry and Molecular Biology, Institute for Biophysical Dynamics, Howard Hughes Medical Institute, University of Chicago, Chicago, IL 60637 USA

## Abstract

*N*^6^-methyladenosine (m^6^A) on mRNAs is critical for various biological processes, yet whether m^6^A regulates drug resistance remains unknown. Here we show that developing resistant phenotypes during tyrosine kinase inhibitor (TKI) therapy depends on m^6^A reduction resulting from *FTO* overexpression in leukemia cells. This deregulated FTO-m^6^A axis pre-exists in naïve cell populations that are genetically homogeneous and is inducible/reversible in response to TKI treatment. Cells with mRNA m^6^A hypomethylation and *FTO* upregulation demonstrate more TKI tolerance and higher growth rates in mice. Either genetic or pharmacological restoration of m^6^A methylation through FTO deactivation renders resistant cells sensitive to TKIs. Mechanistically, the FTO-dependent m^6^A demethylation enhances mRNA stability of proliferation/survival transcripts bearing m^6^A and subsequently leads to increased protein synthesis. Our findings identify a novel function for the m^6^A methylation in regulating cell fate decision and demonstrate that dynamic m^6^A methylome is an additional epigenetic driver of reversible TKI-tolerance state, providing a mechanistic paradigm for drug resistance in cancer.

## Introduction

Leukemia is an aggressive malignancy frequently associated with activating mutations of receptor tyrosine kinases (RTKs), including BCR/ABL, KIT and FLT3 etc.^1–4^ Many tyrosine kinase inhibitors (TKIs) against these mutations have entered the clinic, but rapidly acquired resistance to TKIs represents a major hurdle to successful leukemia treatment. The most commonly cited mechanism is the acquired drug-resistance mutations that impair drug binding or bypass the inhibited RTK signaling.^5,6^ However, these genetic events are insufficient to explain the following scenario where the appearance of TKI resistance is relatively prompt upon drug exposure and the resistant phenotypes are reversible after a “drug holiday”. Also many patients with resistance express exclusively native kinases (e.g., BCR/ABL) or have activated parallel pathways, involving overamplification of oncogenes (e.g., *BCL-2*, *BCL-6*, *AXL* and *MET*).^7–10^ In fact, recent findings have linked acquired TKI resistance to cellular heterogeneity within tumors and dynamic variation in epigenome configurations.^11–13^ It is postulated that the distinct epigenetic patterns in heterogeneous tumor cell populations could generate diversity in the expression of cell fate determination genes that can swiftly evolve through drug selection. However, the delineation of the key epigenetic events in TKI resistance is far from complete.

*N*^*6*^-methyladenosine (m^6^A) is the most common epitranscriptomic modification on mammalian mRNA.^14–16^ It is installed by a methyltransferase complex (e.g., METTL3-METTL14)^17,18^ and can be erased by demethylases (e.g., FTO and ALKBH5).^19,20^ While the precise role of any particular m^6^A residue is unclear,^21^ abundant evidence supports that m^6^A methylation, in general, critically regulates mRNA stability, splicing and/or protein translation,^22–25^ thereby impacting gene expression. Consistently, silencing of m^6^A methyltransferase (e.g., *IME4*, the yeast orthologue of *METTL3*) or knockdown of *FTO* changes m^6^A abundance, re-modeling gene expression profile and/or alternative splicing pattern of transcripts.^26–28^ Despite recent works on roles of m^6^A in various biological processes,^23^ whether and how m^6^A methylation regulates cell fate decisions under TKI selection remain unknown. We hypothesized that, upon exposure to TKIs, the reversible nature of m^6^A methylation allows a set of proliferation/anti-apoptotic oncogenes bearing m^6^A sites to be upregulated, thus helping a subpopulation of cells escape TKI-mediated killing. To test this, we modeled and characterized TKI resistance in distinct leukemia models and directly mapped m^6^A in the transcriptomes of leukemia cells. Our findings demonstrate an intrinsic and inducible FTO-m^6^A axis as a novel marker characterizing the heterogeneous nature of leukemia cells, and a broad defense mechanism by which leukemia cells develop TKI-resistant phenotypes. Our discoveries establish the feasibility to target the FTO-m^6^A axis for prevention/eradication of acquired TKI resistance.

## Results

### TKI-resistant cells survive and proliferate in the absence of targeted RTK signaling

**Fig. 1:**
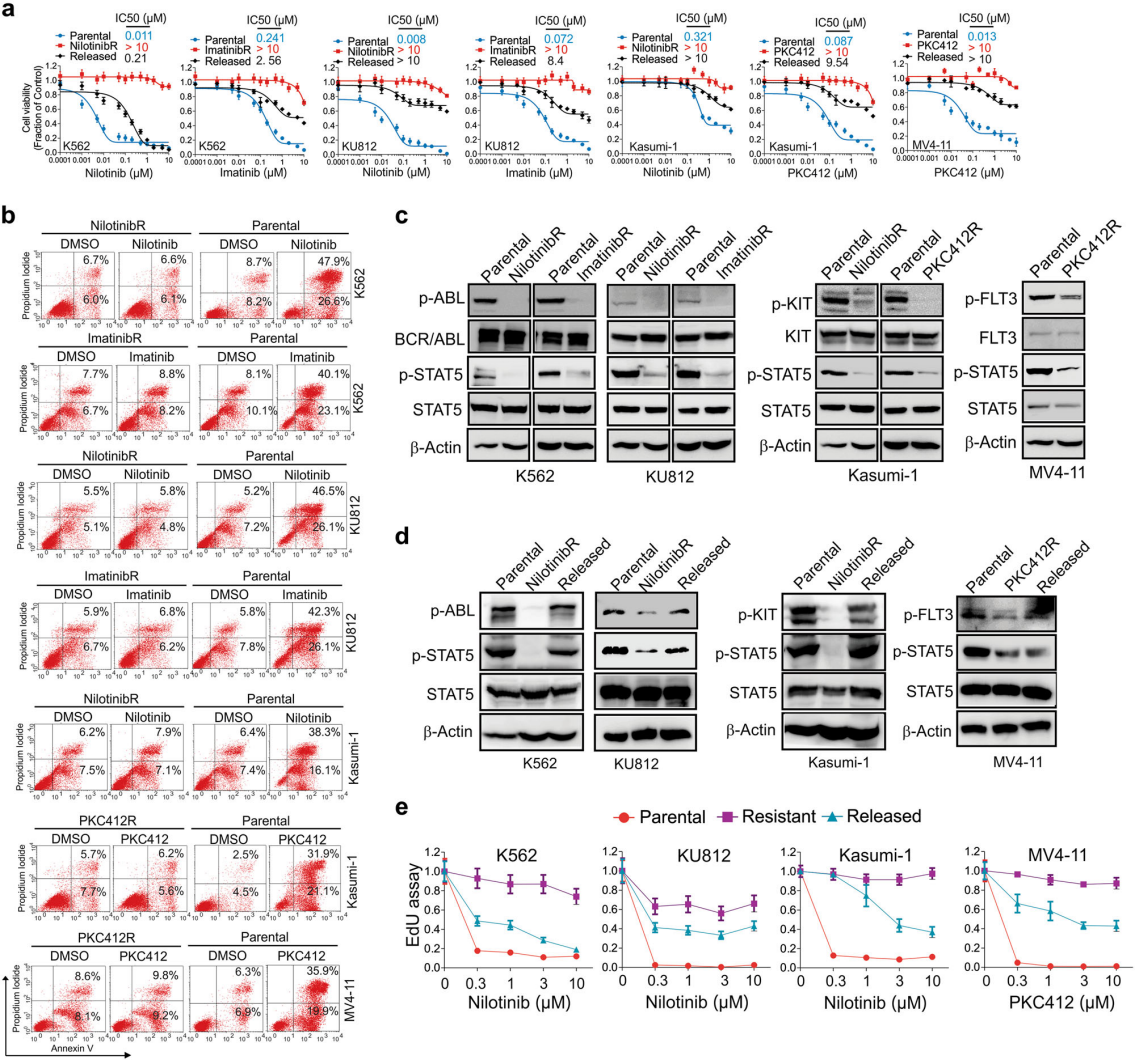
TKI-resistant phenotypes are reversible. **a** CCK-8 assays in parental, resistant and released cells treated with nilotinib, imatinib or PKC412 for 72 h. The data represent two independent experiments with 8 repeats in total. **b** Flow cytometry assays in parental and resistant cells treated with 1 µM nilotinib, imatinib or PKC412 for 72 h. **c** Western blotting of parental and resistant cells. **d** Western blotting of resistant cells cultured in drug-free medium for 15 days. The resistant cells growing in drug-containing medium and the respective parental cells were used as controls. **e** The parental, resistant and released cells were treated with 0.3, 1, 3 or 10 µM indicated drugs for 72 h, incubated with 10 µg/ml EdU for 1–2 h and subjected to flow cytometry for EdU incorporation. Experiments were performed in three biological replicates. In **b**–**d**, data represent three independent experiments

To understand TKI resistance mechanisms, a panel of four representative leukemia cell lines with activating mutations, *BCR/ABL* (K562, KU812), *KIT* (Kasumi-1) and *FLT3* (MV4-11), rendering them sensitive to kinase-targeted therapies were initially exposed to increasing concentrations of representative TKIs, nilotinib, imatinib, or PKC412, until they could grow in medium containing 1 μM of the respective drug. The drug doses were physiologically relevant, which were equivalent to or lower than the peak plasma/serum levels of nilotinib (4 μM), imatinib (5 μM) and PKC412 (1 μM).^29^ To characterize these TKI-selected cells, we measured the survival rate of parental, resistant and released (drug withdrawal for 15 days) cells upon transient exposure to TKIs. As shown in Fig. 1a, the resistant cells displayed IC_50_ values to TKIs several orders of magnitude larger than those exhibited by their parental counterparts. Although all parental controls displayed significant and dose-dependent decreases of cell viability, the resistant cells could proliferate at drug concentrations much larger than the IC_50_ value. Interestingly, the released cells reacquired partial sensitivity to TKIs as supported by a dose-dependent reduction of cell proliferation. When treated with 1 μM TKIs, a dose used to generate resistant cells, the parental control had substantial increases in annexin V/PI positivity, whereas resistant cells remained minimally affected (Fig. 1b). The phosphorylation of BCR/ABL, KIT and FLT3 was present at high levels in parental cells, but barely detectable in resistant cells with a concurrent dephosphorylation of STAT5, a downstream mediator of BCR/ABL, KIT and FLT3 signaling (Fig. 1c). Further, nilotinibR (K562, KU812 and Kasumi-1) and PKC412R (MV4-11) cells rapidly restored the phosphorylation of BCR/ABL, KIT and STAT5 after drug withdrawal (Fig. 1d). Exposure of these released cells to TKIs induced growth arrest supported by a dose-dependent decrease of EdU incorporation which was less pronounced compared to the parental cells (Fig. 1e). Sequencing of the ABL kinase domain in K562 and KU812 cells resistant to imatinib or nilotinib did not identify new mutations (not shown), as has been shown previously.^30^ Therefore, we propose that these resistant cells appear to possess a nongenetic form of TKI resistance.^9,31^

### Transcriptome-wide m^6^A sequencing and expression characterizations of differentially expressed genes in TKI resistant cells

**Fig. 2:**
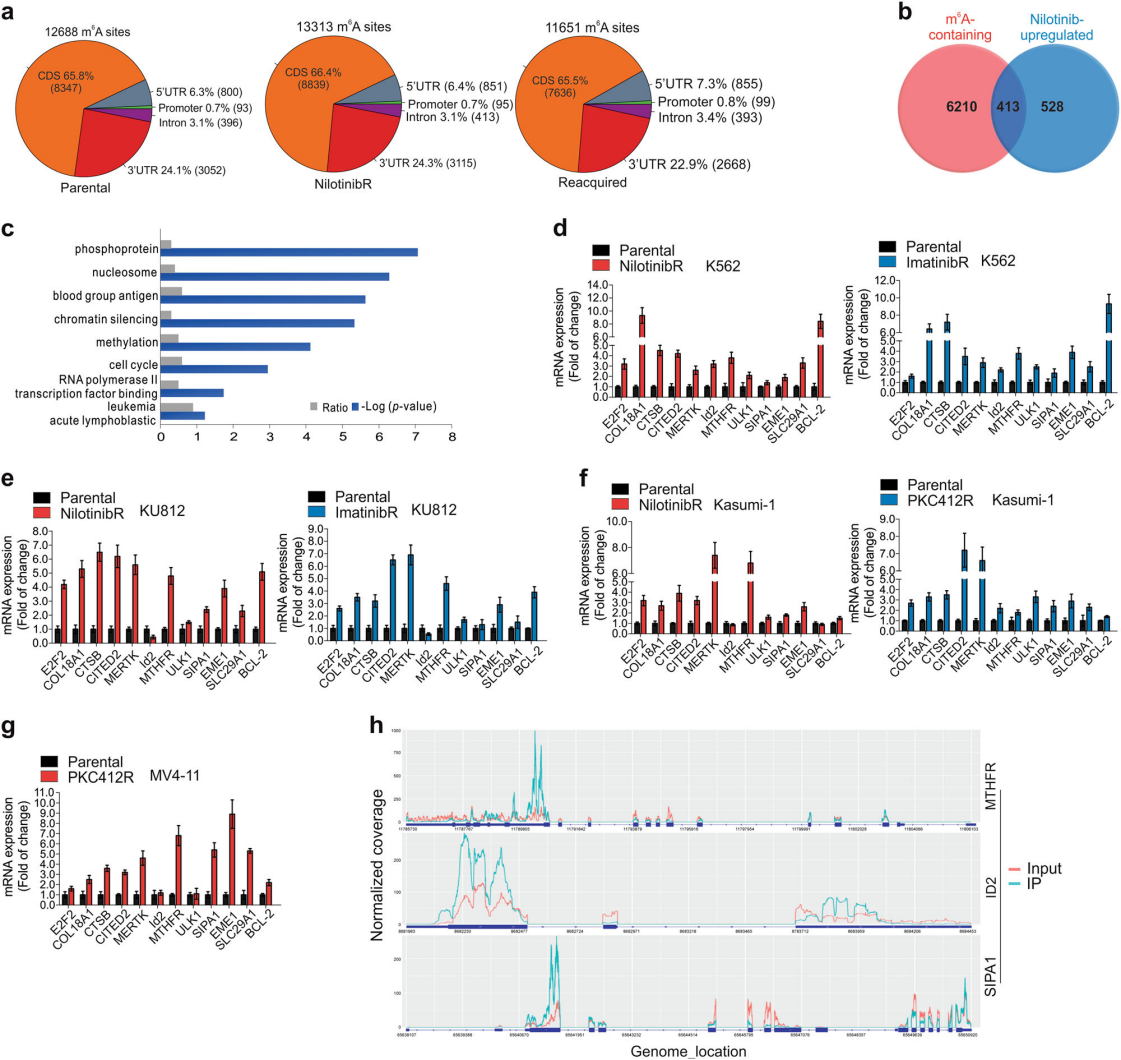
Characterization of the m^6^A-bearing genes in TKI resistant cells. **a** Pie chart presenting fractions of m^6^A peaks in different transcript segments. CDS, coding sequence; UTR, untranslated region. **b** Venn diagram illustrating the number of overlapped genes between m^6^A containing and differentially-expressed gene profiles. **c** Enrichment scores for Gene Ontology (GO) categories in overlapped genes. The –log (*p* value) axis indicates the statistical significance of the functions to the dataset. **d**–**g** qPCR measuring the expression levels of indicated genes in TKI resistant *vs* parental K562, KU812, Kasumi-1 and MV4-11 cells. Data represent three independent experiments. **h** Coverage plot of m^6^A IP and input reads (without smoothing the curve). IP, immunoprecipitation; IP, blue; input, red. See also Figures S1 and S2

As acquired TKI resistance is frequently associated with aberrantly expressed bona fide oncogenes, we performed gene expression profiling in K562 cells resistant to nilotinib (nilotinibR), a second-generation TKI^32^ with the emergence of drug resistance.^33^ There were 941 upregulated (Table S1a) and 1214 downregulated (Table S1b) genes identified in nilotinibR *vs* parental K562 cells (fold change ≥ 1.5). While multiple mechanisms could mediate such differential expression, we examined m^6^A mRNA methylation, which is a reversible and dynamic process in controlling gene expression,^23^ given the dynamic and reversible nature of the TKI resistant phenotypes^34^ (ref. Figure 1a, d, e). To investigate the distribution features of m^6^A methylation in response to drug selection, we conducted m^6^A sequencing (m^6^A-seq) in K562 parental, nilotinibR cells and those that had reacquired sensitivity to nilotinib. In total, 12,000–15,000 m^6^A-enriched regions/m^6^A peaks on mRNA of ~7000 genes were identified across the transcriptome under each condition (Table S1c-e). Consistent with previous reports,^15,27,28,35,36^ the majority of m^6^A sites (>60%) were enriched in the coding sequence (CDS) region of mRNAs, with 23–25% in 3′UTR and 5–7% in 5′UTR (Fig. 2a). About 60% of the peak sequence contained GGACU/GAACU/AAACU/AGACU, and the top hits of sequences across the conditions were shown in Fig. S1a. Notably, these m^6^A sites appear to be largely static across diverse conditions, in agreement with the previous report that m^6^A motifs ([G/A/U][G > A]m^6^AC[U > A/C]) are relatively conservative.^27,35^

Next, we overlapped genes in expression profiles with transcripts having m^6^A sites identified by m^6^A-seq in K562 parental cells. Because not all molecules of a specific mRNA are expected to be methylated, we used a low fold-change threshold of 1.5, although at this level it may introduce false positive mRNAs. A large portion of genes, including 413 upregulated (~44%, 413/941; Fig. 2b; Table S1f) and 579 downregulated (~48%, 579/1214; Fig. S1b; Table S1g), displayed m^6^A methylation peaks. This was further verified by comparing a reported m^6^A signature in HEK293 cells,^27^ where 263 upregulated (~28%, 263/941) and 333 downregulated (~27%, 333/1214; Fig. S1c and d; Table S1h and i) genes bore m^6^A sites. These m^6^A-bearing genes are involved in many cellular processes, including nucleic acid/mRNA metabolic processes, methylation, mRNA splicing, translation/protein biosynthesis, cell cycle and proliferation etc. In addition, m^6^A peaks were mapped to numerous genes involved in leukemia diseases (Fig. 2c; Fig. S1e-g; Table S1j and k).

We then selected the following genes, *E2F2*, *COL18A1*, *CTSB*, *CITED2*, *MERTK*, *ID2*, *MTHFR*, *ULK1*, *SIPA1*, *EME1* and *SLC29A1*, for further investigations, because these genes were upregulated, although not necessarily to the highest level, in resistant cells detected by expression profiling, and carried m^6^A sites identified by overlapping analysis. Also, they are either well-established resistant genes and/or annotated to have a role in sustaining cancer cell survival and proliferation. We initially utilized qPCR to confirm their upregulation in K562, KU812, Kasumi-1 and MV4-11 cells resistant to different TKIs (Fig. 2d–g). To broaden m^6^A implications in TKI resistance, we used a well-known TKI resistant gene, *BCL-2*,^37^ as an example and confirmed its upregulation in resistant cells (Fig. 2d–g). We then verified the presence of m^6^A sites in these genes in m^6^A-seq and presented examples of m^6^A peaks for *MTHFR*, *ID2*, and *SIPA1* in parental cells (Fig. 2h). Notably, although it was a relatively small number, we did observe differentially methylated genes in nilotinibR or nilotinib-resensitized cells compared to parental cells (Fig. S2a, b; Table S1l-q). In line with this, a small fraction of new m^6^A peaks emerged in nilotinibR cells, with additional m^6^A peaks that were lost in nilotinibR re-appeared when cells re-acquired nilotinib sensitivity (Table S1r, s). In addition, the majority of genes, including some of the aforementioned targets, did not show significant differential methylation (Fig. S2c–e), and most m^6^A peaks were conserved across nilotinib treatment (Table S1t). Given that FTO upregulation was impaired and m^6^A demethylation was rescued in K562 nilotinib-resensitized cells (Fig. S2f and g), these findings support the idea that the development of TKI resistant phenotypes could mainly involve changes in m^6^A abundance.

### The m^6^A levels are decreased through *FTO* upregulation in TKI resistant cells

**Fig. 3:**
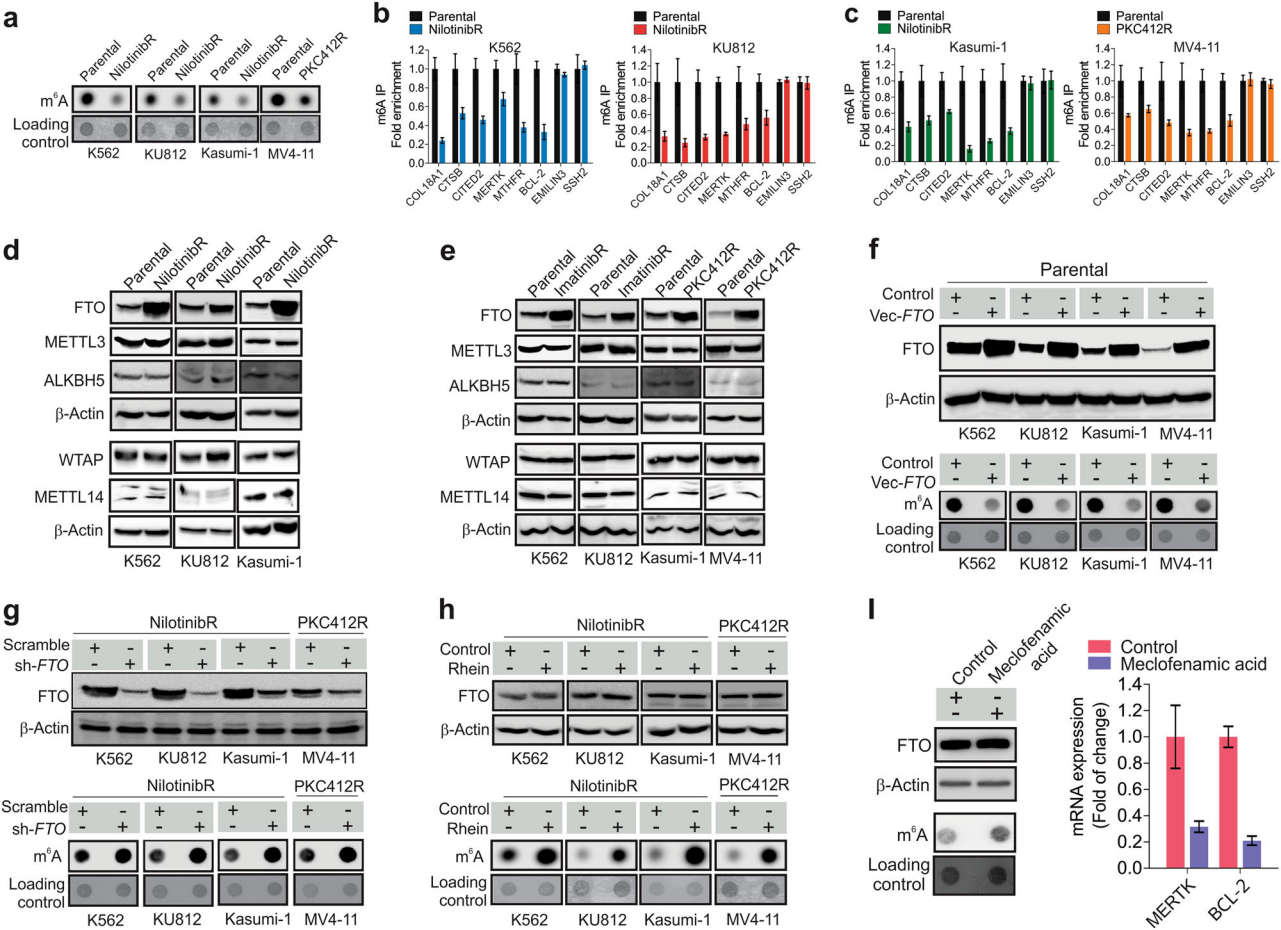
FTO upregulation mediates m^6^A demethylation in TKI resistant cells. **a** Dotblotting for mRNA from parental and resistant cells using anti-m^6^A antibody. **b**, **c** The eluted mRNA from anti-m^6^A immunoprecipitates in parental and resistant cells was subjected to cDNA synthesis, and gene expression was determined by qPCR. **d**, **e** Western blotting of parental and resistant cells. **f** The parental cells were transfected with *FTO* expression or control vectors for 48 h. **g**, **h** The resistant cells were transfected with *FTO* shRNA or scramble vectors (**g**) or treated with 25 µM rhein (**h**) for 48 h. **i** K562 nilotinibR cells were treated with 50 µM meclofenamic acid for 48 h. Left, Western blotting for FTO protein expression and dotblotting for m^6^A abundance; right, qPCR for gene expression. Data represent three independent experiments. In **f**, **g** and **h**, the cell lysates were subjected to Western blotting (upper) and the mRNA for dotblotting using anti-m^6^A (lower); sh, shRNA; Vec, vector; Data represent three independent experiments; Data are mean ± SD; *p *<* 0.05, **p *<* 0.01. See also Figure S3 and Table S2

To assess m^6^A changes in resistant cells, we performed dotblotting using anti-m^6^A antibody, which specifically and quantitatively measures m^6^A abundance,^28,38^ in mRNA from parental, nilotinibR and PKC412R cells. These experiments identified a notable decrease of global m^6^A abundance in resistant *vs* parental cells (Fig. 3a). We applied a read count-based analysis method, in which we directly calculated m^6^A-peak fold enrichment from normalized read counts mapped to peak region (Table S2a, b) and compared summary statistics across experimental conditions. The data showed that the overall peak fold enrichment decreased in resistant *vs* parental cells, but increased in resensitized *vs* resistant cells (Fig. S3a). Importantly, the m^6^A-IP followed by qPCR using primers covering putative m^6^A sites revealed an appreciable reduction of m^6^A methylation in resistant cells on specific genes, such as *COL18A1*, *CTSB*, *CITED2*, *MERTK*, *MTHFR* and *BCL-2* (Fig. 3b, c), further verifying them as methylated genes identified by m^6^A-seq. As negative controls, we performed qPCR using primers outside m^6^A motifs and detected only background signal (Fig. S3b). We also checked m^6^A-containing genes (e.g., *EMILIN3*, *SSH2*) that were not affected by TKIs, and the m^6^A-IP followed by qPCR failed to detect obvious changes (Fig. 3b, c). These results support that chronic exposure to TKIs induces global and gene specific reduction of m^6^A methylation.

To elucidate the mechanisms underlying m^6^A hypomethylation in TKI resistant cells, we examined changes in mRNA and protein levels of FTO, ALKBH5, METTL3, METTL14 and WTAP, which cooperatively regulate m^6^A methylation.^14,17–19,23,39^ In contrast to the changes in m^6^A abundance, the protein expression of FTO, but not ALKBH5, METTL14, WTAP nor METTL3, was substantially increased in K562, KU812 and Kasumi-1 nilotinibR cells (Fig. 3d). Such nilotinib-mediated FTO upregulation was also found in imatinibR or PKC412R cells (Fig. 3e), suggesting that FTO upregulation is broadly relevant in response to TKI exposure. However, the *FTO* mRNA expression in resistant cells did not show substantial changes (Fig. S3c). In support of FTO as an mRNA demethylase, enforced *FTO* expression (Fig. 3f, upper) in parental cells expressing relatively low FTO decreased m^6^A methylation (Fig. 3f, lower). In contrast, using a mixture of three shRNA vectors targeting different regions of the *FTO* gene, we knocked down *FTO* (Fig. 3g, upper) in resistant cells carrying *FTO* upregulation, and observed an increase of m^6^A abundance (Fig. 3g, lower). Consistently, treatment with FTO inhibitor rhein^40^ enhanced m^6^A methylation with barely detectable changes in FTO protein expression (Fig. 3h). Because potential off-target effects of rhein possibly exist, we examined another FTO inhibitor, meclofenamic acid,^41^ and obtained similar results (Fig. 3i). These findings support the idea that the altered m^6^A methylome could be attributable to the FTO changes in response to TKI treatment.

### m^6^A demethylation by FTO leads to overexpression of survival and proliferation genes

**Fig. 4:**
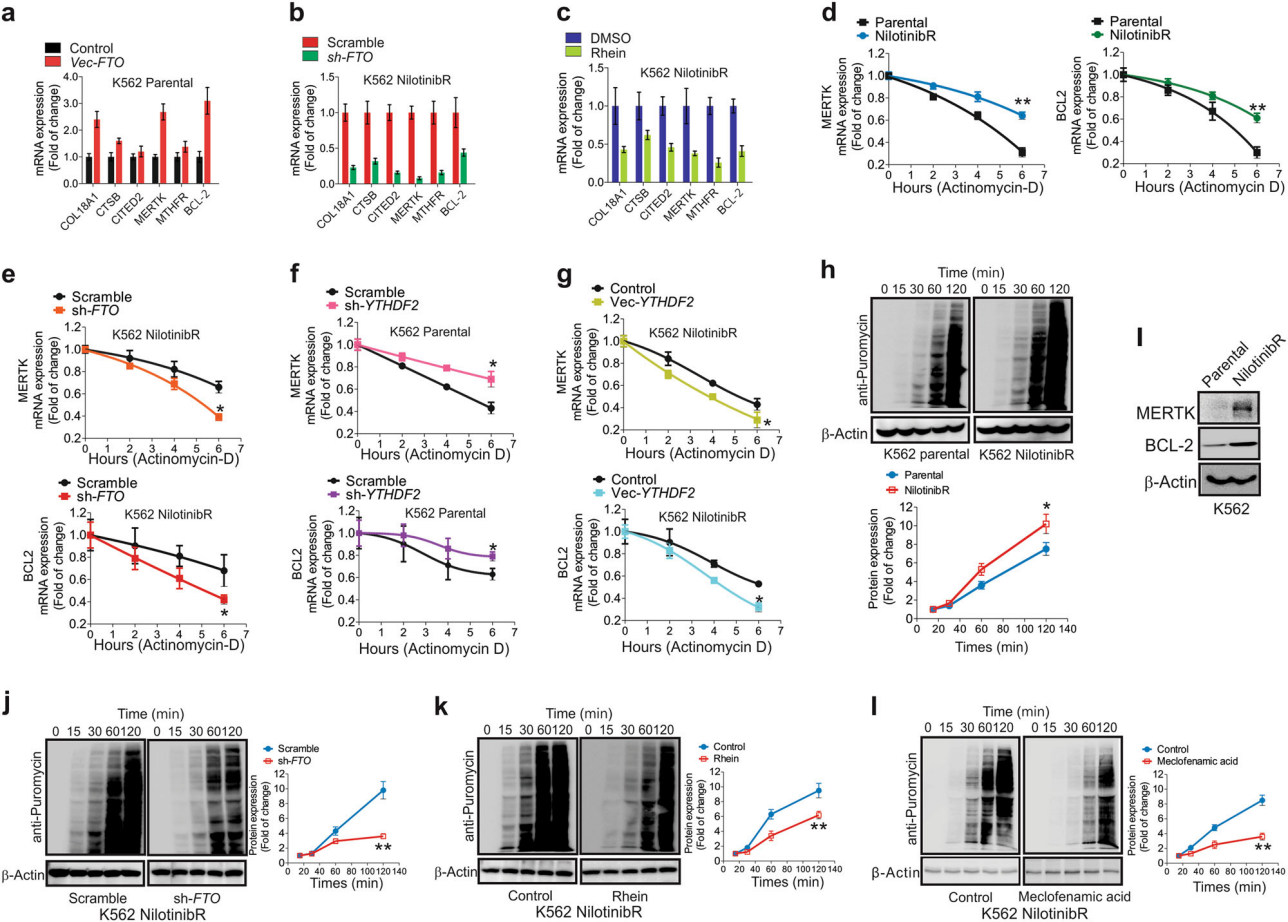
FTO-demethylated m^6^A promotes gene expression at post-transcriptional levels. **a**, **b** qPCR of K562 parental cells transfected with *FTO* expression or control vectors (**a**) or nilotinibR cells transfected with *FTO* shRNA or scramble vectors (**b**) for 48 h. **c** qPCR of K562 nilotinibR cells exposed to 25 µM rhein for 48 h. **d** qPCR of parental and resistant K562 cells treated with actinomycin-D (5 μg/ml) for the indicated time points. The gene expression was normalized to *GAPDH*. **e** qPCR of K562 nilotinibR cells transfected with *FTO* shRNA or scrambled vectors for 48 h followed by 5 μg/ml actinomycin-D treatment for the indicated time points. The gene expression was normalized to *GAPDH*. **f**, **g** K562 parental cells were transfected with *YTHDF2* shRNA (**f**) or nilotinibR cells with *YTHDF2* expression (**g**) vectors for 48 h followed by 5 μg/ml actinomycin-D treatment for the indicated time points. The gene expression was determined by qPCR and normalized to *GAPDH*. **h** Parental and resistant K562 cells were treated with 200 nM puromycin for the indicated time points. **i** Western blotting of K562 parental and resistant cells. **j**–**l** K562 nilotinibR cells were transfected with *FTO* shRNA vectors (**j**), treated with 25 µM rhein (**k**) or 50 µM meclofenamic acid (**l**) for 48 h followed by 200 nM puromycin treatment for the indicated time points. In **h** and **j**–**l**, graphs are the quantification of Western blotting results normalized to β-actin; Data represent three independent experiments; Data are mean ± SD; *p < 0.05, **p < 0.01. See also Figure S3

To explore the impact of m^6^A hypomethylation on gene expression, we transiently expressed *FTO* in K562 parental cells, and inactivated *FTO* in K562 nilotinibR cells by either gene knockdown or rhein. These experiments revealed that enforced *FTO* expression increased, whereas FTO inactivation impaired, the expression of *COL18A1*, *CTSB*, *CITED2*, *MERTK*, *MTHFR* and *BCL-2* (Fig. 4a–c). Because m^6^A methylation influences the lifetime of mRNA,^22^ to suppress mRNA synthesis, we exposed K562 parental and nilotinibR cells to actinomycin-D, a transcriptional inhibitor. As shown in Fig. 4d, the mRNAs of *MERTK* and *BCL-2* in resistant cells displayed a lower degradation rate compared to parental cells, consistent with an earlier report showing that m^6^A demethylation enhances RNA stability.^22^ Conversely, *FTO* knockdown followed by actinomycin-D treatment propelled the degradation of *MERTK* and *BCL-2* mRNAs (Fig. 4e). These results support that the mRNAs of *MERTK* and *BCL-2* in resistant cells become more stable, in agreement with the finding that m^6^A methylation predominantly and directly decreases transcript stability.^42^

FTO has recently been shown to also demethylate m^6^A_m_ near the cap of certain mRNAs,^43^ which reduces the target transcript stability. Based on this study,^43^ the elevated FTO levels in resistance cells could possibly decrease the m^6^A_m_ abundance leading to reduced mRNA stability, which was opposite to what we observed. Furthermore, when comparing the density changes of transcriptome-wide m^6^A-seq reads in mRNA of resistant *vs* parental cells, we found that most changes in m^6^A peaks came from IncRNA as well as CDs and 3′UTR with less profound changes at 5′UTR close to cap, where m^6^A_m_ was located (Fig. S3d). In addition, we found that the overall abundance of m^6^A was at least 15-fold higher than m^6^A_m_ in leukemia cells. While cap m^6^A_m_ might play functional roles in mRNA stability in other systems,^43^ we believed the major effects on transcript stability observed in K562 nilotinibR cells were mediated through demethylation of internal m^6^A by FTO, which was consistent with a recent report in AML.^28^ An m^6^A reader protein YTHDF2 was known to reduce the stability of mRNAs containing m^6^A.^22^ We transfected K562 parental or resistant cells with *YTHDF2* shRNA or expression vectors, respectively, and found that *YTHDF2* knockdown augmented, whereas enforced *YTHDF2* expression decreased, the mRNA stability of *BCL-2* and *MERTK* (Fig. 4f, g), which was inversely correlated with *FTO* loss/gain of functions (ref. Figure 4d, e).

Finally, because m^6^A methylation also regulates mRNA translation efficiency,^25,44^ we employed SUnSET,^45^ a nonradioactive method, to monitor protein synthesis in the presence of puromycin, a protein synthesis inhibitor. Western blotting using anti-puromycin antibody showed that more puromycin was detected in proteins from resistant than parental cells when normalized to β-actin (Fig. 4h), suggesting a higher rate of protein synthesis that was also supported by higher protein levels of MERK and BCL-2 in resistant than in parental cells (Fig. 4i). When FTO was inactivated in resistant cells either by gene knockdown (Fig. 4j) or by its inhibitors (Fig. 4k, l), the integration of puromycin into newly synthesized proteins was notably decreased. These data support the idea that m^6^A demethylation by FTO maintains oncogenic overexpression partially through its impacts on mRNA stability which leads to increased protein synthesis.

### Demethylation of m^6^A occurs in second-line TKI resistance

Analogous to first-line therapy, the second-line treatment with more recently developed TKIs (e.g., nilotinib) achieves an initial response, but further development of drug resistance impedes their therapeutic benefits.^46^ To elucidate the mechanisms responsible for this resistance, we first generated imatinib-resistant K562 and KU812 cells, followed by exposure of them to nilotinib starting with low doses with subsequent stepwise increase in their concentrations. Consistently, the imatinibR cells were sensitive to nilotinib initially and the BCR/ABL signaling was dose-dependently suppressed leading to cell growth arrest (not shown). Then these cells regained the capability to propagate, and the second-line resistant phenotypes (K562^ImR+NiR^, KU812^ImR+NiR^) were considered to be established when the imatinibR cells could grow in medium containing 1 µM nilotinib. Upon transient exposure to nilotinib, the cell proliferation rate of first-line imatinibR cells was dose-dependently reduced, but K562^ImR+NiR^ and KU812^ImR+NiR^ cells were insensitive to nilotinib with IC_50_ values several orders of magnitude greater (Fig. S4a). Although they survived and expanded in the presence of nilotinib, the targeted kinase signaling in K562^ImR+NiR^ and KU812^ImR+NiR^ cells remained inactivated, as the phosphorylation of BCR/ABL and STAT5 was barely detectable compared to parental cells (Fig. S4b), supporting a BCR/ABL-independent mechanism. Mechanistically, the expression of FTO, but not ALKBH5 and METTL3, was highly elevated (Fig. S4c), and the amount of global m^6^A was notably reduced (Fig. S4d), in K562^ImR+NiR^ and KU812^ImR+NiR^ cells. The m^6^A-IP followed by qPCR revealed the decreased m^6^A abundance on mRNA of *MERTK* and *BCL-2* (Fig. S4e), but the qPCR without m^6^A-IP detected their upregulation (Fig. S4f). Further, exposure of K562^ImR+NiR^ and KU812^ImR+NiR^ to rhein increased m^6^A methylation (not shown) accompanied by *MERTK* and *BCL-2* downregulation (Fig. S4g). These findings support the idea that FTO-driven m^6^A demethylation may sustain the activity of target genes required for second-line TKI resistance.

### Establishment of TKI resistance requires the *FTO* gene

**Fig. 5:**
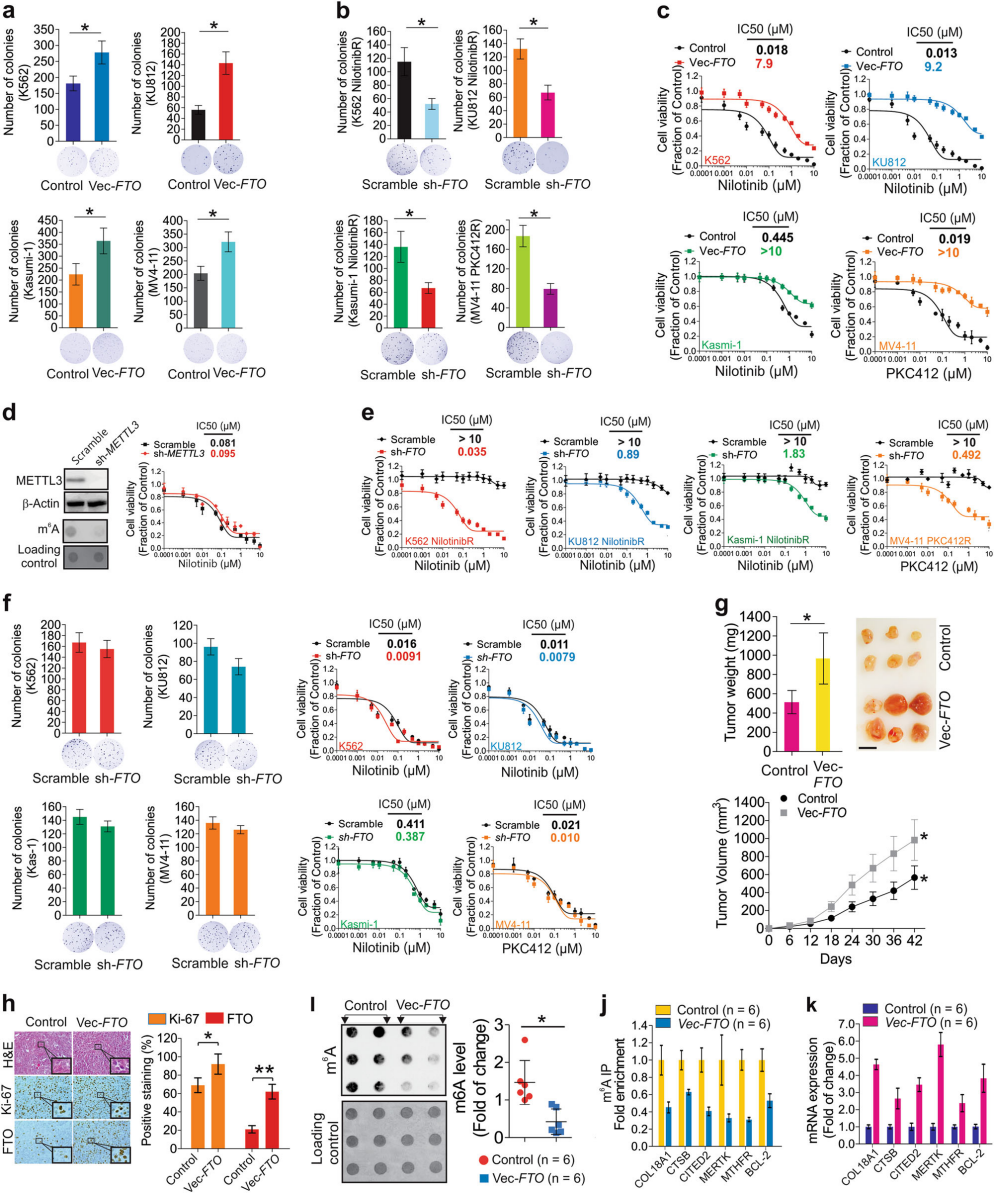
FTO promotes TKI resistance. **a**, **b** Parental cells were transiently transfected with *FTO* expression (**a**) or resistant cells with *FTO* shRNA (**b**) vectors and subjected to colony assays in medium free from nilotinib. **c** CCK-8 assays for parental cells transfected with *FTO* expression or control vectors for 24 h followed by nilotinib or PKC412 treatment for another 48 h. **d** Left, Western blotting or dotblotting of K562 parental cells transfected with *METTL3* shRNA or scramble vectors for 48 h. Data represent three independent experiments; right, CCK-8 assays for K562 parental cells transfected with *METTL3* shRNA or scramble vectors for 24 h followed by nilotinib treatment for additional 48 h. **e** CCK-8 assays for resistant cells transfected with *FTO* shRNA or scramble vectors for 24 h followed by nilotinib or PKC412 treatment for another 48 h. **f** The parental cells were transfected with *FTO* shRNA or scramble vectors for 24 h and subjected to colony assays in drug-free medium (left) or CCK-8 assays (right) after nilotinib treatment for another 48 h. **g** External view of tumors, and graphs show the quantification of tumor weight or tumor volume. **h** Left, representative images of IHC and H&E staining. Window stands for the enlarged cells; right, graph is the quantification of IHC staining. **i** Left, representative images of m^6^A dotblotting from tumors; right, graph is the quantification of blot intensities. **j** IP by anti-m^6^A antibody was performed in mRNA extracted from tumors. The eluted mRNA was subjected to cDNA synthesis followed by qPCR for gene expression. **k** qPCR for gene expression in mRNA extracted from tumors. Data in colony assays represent two independent experiments with four duplicates; Data in CCK-8 assays represent two independent experiments with 8 repeats; In **j**, **k**, *n* = 6 tumors/group; Data are mean ± SD; *p *<* 0.05, **p *<* 0.01. See also Figures S4, S5 and S6

Next, we sought to determine whether resistance is induced by TKI treatment or, alternatively, if a subpopulation of unexposed cells is already drug-tolerant. In support of the latter possibility, we performed colony assays and isolated single clones from naïve cell lines, K562 and Kasumi-1, and primary cells from CML and AML patients. As shown in Fig. S5A and S5B, the sizes of clones were different, revealing the phenotypic heterogeneity of leukemia cell populations. Although there was no detectable difference regarding the protein expression and phosphorylation of BCR/ABL and KIT (Fig. S5c, d), higher FTO with lower m^6^A was present in large compared to small clones (Fig. S5c–h). Further, the *FTO* levels and colony sizes had a positive correlation (Fig. S5i), implying that *FTO* overexpression possibly originates from the large clones. We also noted that large clones exhibited higher levels of *MERTK* and *BCL-2* (Fig. S6a, b), and, importantly, higher intrinsic tolerability to nilotinib (Fig. S6c, d). These results suggest that, in response to TKI selection, the large clones could possibly have more chance to survive and proliferate than small clones. In support of this, *FTO* overexpression in parental cells with lower FTO (ref. Figure 3d, e) increased the colony number (K562, 181 ± 23 *vs* 278 ± 36; Kasumi-1, 224 ± 45 *vs* 364 ± 54; KU812, 58 ± 6 *vs* 141 ± 22; MV4-11, 204 ± 26 *vs* 321 ± 37; Fig. 5a). Conversely, *FTO* knockdown in resistant cells with higher FTO (ref. Figure 3d, e) reduced the colony number (K562, 115 ± 21 *vs* 52 ± 8; Kasumi-1, 136 ± 26 *vs* 67 ± 9; KU812, 131 ± 11 *vs* 62 ± 7; MV4-11, 187 ± 22 *vs* 79 ± 11; Fig. 5b). In response to TKIs, *FTO* overexpression desensitized parental cells to nilotinib or PKC412 with notably higher IC_50_ values (Fig. 5c), in line with the effects of m^6^A demethylation mediated by *METTL3* knockdown (Fig. 5d). In contrast, *FTO* down-modulation rendered resistant cells more sensitive to nilotinib or PKC412, as supported by the decreased cell proliferation rate and the lower IC_50_ (Fig. 5e). As a negative control, we also knocked down *FTO* in parental cells, and observed only a trend toward reduced colony number and enhanced TKI sensitivity (Fig. 5f), which possibly resulted from non-selection for FTO-dependence as with the resistant cells.

Finally, to explore the oncogenic potential of *FTO* in vivo, we engrafted nude mice with parental K562 cells overexpressing *FTO*. Although tumor incidence was similar (6/6, 100%), ectopic *FTO* expression led to a more aggressive tumor growth, as indicated by a larger tumor volume (control, 566 ± 130 mm^3^; *FTO*, 983 ± 226 mm^3^), higher tumor weight (control, 513 ± 121 mg; *FTO*, 966 ± 265 mg) and higher tumor cell proliferation supported by more Ki-67 and H&E-stained cells (Fig. 5g, h), consistent with the recent discovery of FTO as an oncogene in leukemia.^28^ The molecular characterization revealed that *FTO* overexpression led to a decrease of global (Fig. 5i) and gene specific (Fig. 5j) m^6^A methylation, but an upregulation of *COL18A*, *CTSB*, *CITED2*, *MTHFR*, *MERTK* and *BCL-2* in tumors (Fig. 5k). Collectively, these findings support that rewired signaling programs driven by *FTO* upregulation regulate leukemia cell fate upon exposure to TKIs.

### Pharmacological and genetic intervention of the FTO-m^6^A axis eradicates TKI resistant cells in vitro and in vivo

**Fig. 6:**
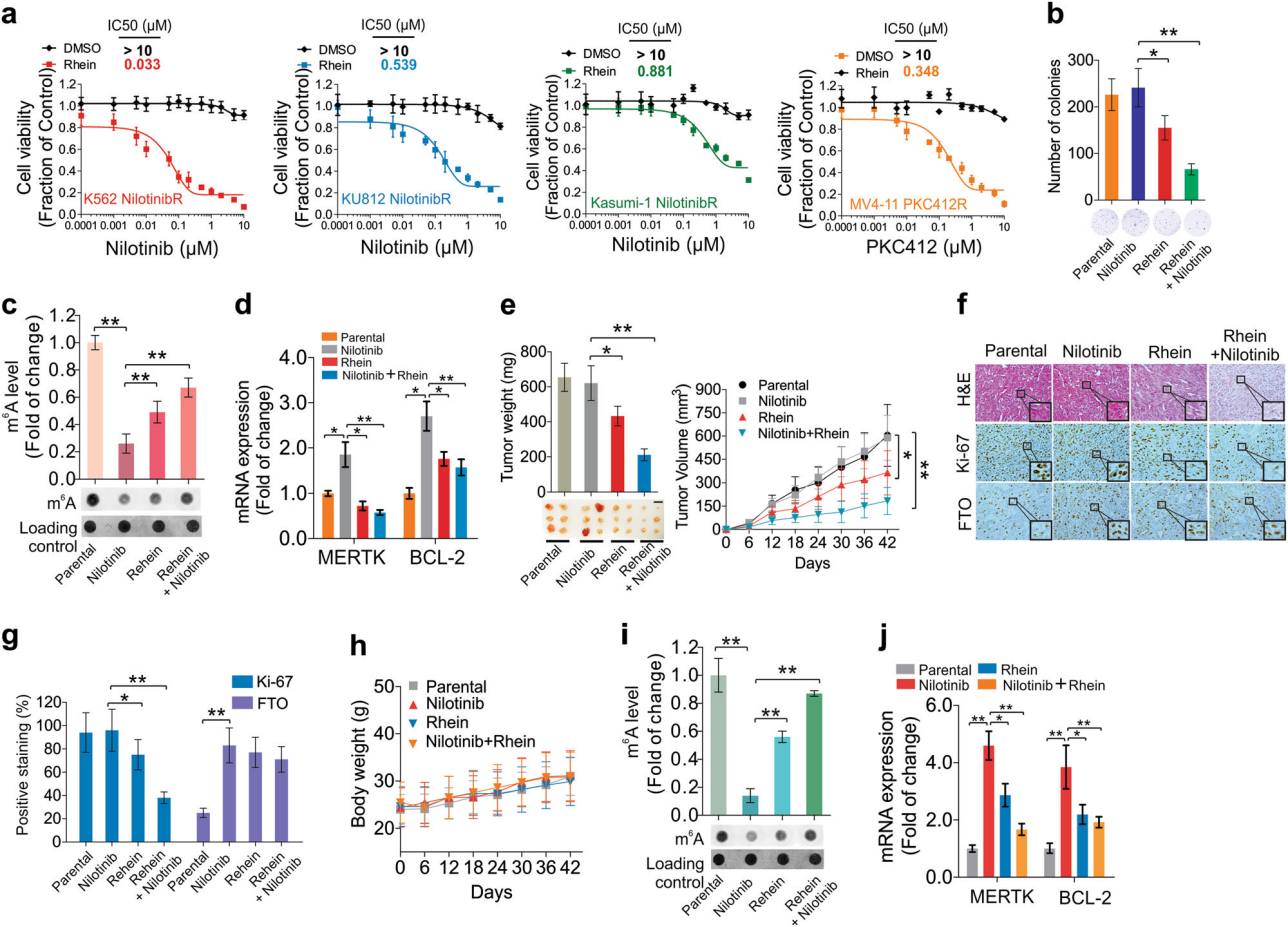
Pharmacological deactivation of FTO sensitizes TKI resistant cells to TKI in vitro and in vivo. **a**, **b** CCK-8 (**a**) or colony-forming (**b**) assays for resistant cells treated with 25 µM rhein for 6 h followed by co-treatment with the FTO inhibitor plus varying concentrations of nilotinib for 72 h. The combination effects were normalized to DMSO only. **c**, **d** Dotblotting (**c**) or qPCR (**d**) of K562 nilotinibR cells treated with 25 µM rhein for 6 h followed by 1 µM nilotinib treatment for another 48 h. **e** Left, the images are the external view of tumors and the measurement of xenograft tumor weight; right, growth curve indicates the tumor volume. **f** Representative images of H&E and IHC staining of tumor sections. Window stands for the enlarged cells. **g** Graphs are the quantification of IHC-stained tumor sections. **h** The graph illustrates the average body weight of tumor-bearing mice. **i** Representative images of m^6^A dotblotting and the quantification of dot intensities in tumors. **j** qPCR for expression of indicated genes in tumors. Data in CCK-8 assays represent two independent experiments with 8 repeats; Data in colony assays represent two independent experiments with four repeats in total; In **e**–**j**, *n* = 6 tumors/ group; Data are mean ± SD; *p *<* 0.05, **p *<* 0.01. See also Figure S7

The requirement of FTO-dependent m^6^A demethylation for resistance could yield a therapeutic opportunity to prevent development of TKI resistant phenotypes. Indeed, exposure to 25 µM rhein induced more pronounced inhibition of cell proliferation in nilotinibR (K562, K812, Kasumi-1) and PKC412R (MV4-11) cells than in parental controls (Fig. S7a); consistent effects were also observed with meclofenamic acid treatment (Fig. S7b). As *FTO* knockdown sensitized resistant cells to TKI treatment (ref. Figure 5e), dual inhibition of RTKs and FTO might be more effective in killing leukemia, particularly the relapsed/refractory disease. We therefore examined the efficacy of rhein-TKI combination therapy. For the in vitro examination, the nilotinibR and PKC412R cells were exposed to 25 µM rhein for 6 h initially to suppress FTO activity, followed by nilotinib treatment for additional 72 h. We found that rhein combination with nilotinib or PKC412 was more effective at lowering cell viability and IC_50_ values (Fig. 6a), and at suppressing colony formation than single-agent treatment (Fig. 6b); similar results were also observed with meclofenamic acid treatment (Fig. S7c, d). Molecular characterization showed that nilotinib-mediated m^6^A hypomethylation was rescued (Fig. 6c), but nilotinib-upregulated expression of *MERTK* and *BCL-2* was impaired (Fig. 6d), by rhein treatment. Notably, when resistant cells were exposed to 10 µM danthron, which was structurally similar to rhein, but an inactive compound for FTO inhibition,^40^ no obvious changes in cell proliferation, colony formation, m^6^A abundance and expression of *BCL-2* and *MERTK* were observed. Importantly, danthron did not sensitize K562 nilotinibR cells to nilotinib (Fig. S7e–h), supporting a specific role of FTO in TKI resistance.

To examine these effects in vivo, we engrafted nude mice with K562 nilotinibR cells. To retain the resistance phenotypes, we continuously administrated 1 mg/kg of nilotinib after cell inoculation by intraperitoneal injection twice a week until the tumor volume approached 10 mm^3^. Randomized groups were treated with suboptimal doses of either nilotinib (5 mg/kg), rhein (10 mg/kg) alone or both. Although continuous administration of nilotinib did not suppress, and rhein itself marginally slowed down resistant tumor growth, the combination therapy resulted in a greater regression of tumor growth, as demonstrated by the smallest tumor volume (parental, 604 ± 199 mm^3^; nilotinib, 589 ± 145 mm^3^; rhein, 364 ± 141 mm^3^; nilotinib plus rhein, 184 ± 87 mm^3^) and lowest tumor weight (parental, 654 ± 80 mg; nilotinib, 621 ± 99 mg; rhein, 433 ± 56 mg; nilotinib plus rhein, 211 ± 34 mg; Fig. 6e). H&E and IHC staining with Ki-67 showed that tumor formation was more significantly suppressed by nilotinib plus rhein (Fig. 6f, g). No drug side effects were observed based on the lack of obvious body weight loss in these mice (Fig. 6h). Mechanistic investigations revealed an increase of the overall mRNA m^6^A methylation (Fig. 6i) and a downregulation of *MERTK* and *BCL-2* (Fig. 6j). These data support the potential of the targeted combination regimens to enhance the therapeutic index of current and emerging TKIs.

### The FTO-m^6^A axis allows primary leukemia cells to survive through nilotinib selection

**Fig. 7:**
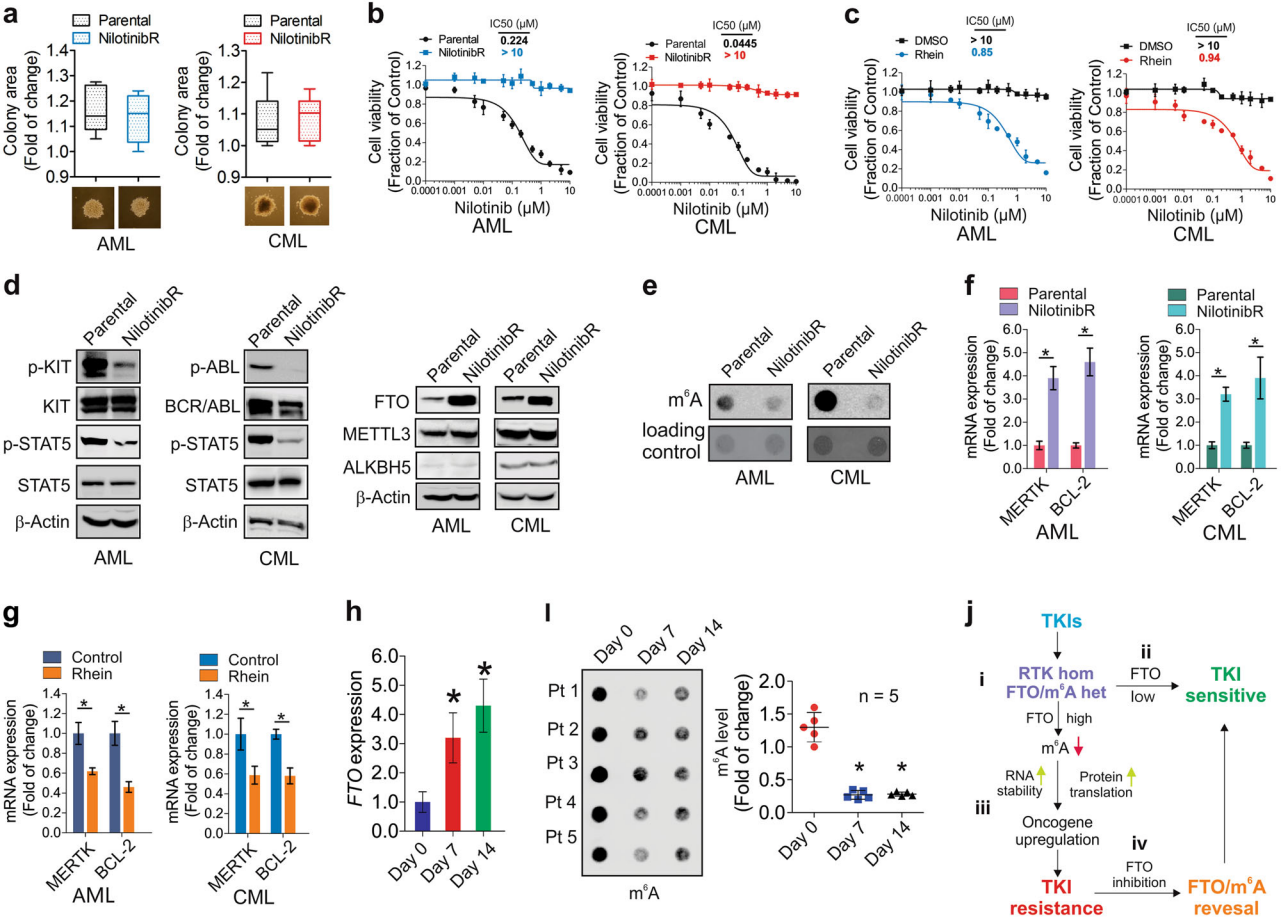
Clinical implications of FTO-mediated m^6^A demethylation in leukemia. **a** Upper, graphs are the quantification of clone sizes (*n* = 5); lower, representative images of parental and resistant clones (*n* = 5) from primary cells of an AML or CML patient. **b** CCK-8 assays in AML and CML parental and resistant clones treated with nilotinib for 72 h. **c** CCK-8 assays in AML and CML resistant clones incubated with 25 µM rhein for 6 h followed by nilotinib treatment for another 72 h. **d** Western blotting of AML and CML clones. The protein lysates are the pool of 5 clones. **e** Dotblotting of AML and CML parental and resistant clones (pool of 5 clones). **f** qPCR of AML and CML parental and resistant clones. **g** qPCR of AML and CML resistant clones (*n* = 5) treated with 25 µM rhein for 48 h. **h** qPCR of RNA extracted from AML patients (*n* = 14) receiving nilotinib therapy. **i** Dotblotting of RNA extracted from nilotinib-treated AML patients (*n* = 5). Left, the image of m^6^A dotblotting; right, graph indicates the quantification of dot intensities. **j** Schematic model illustrating the role of the FTO-m^6^A axis in mediating TKI resistance. (i) The levels of FTO expression/m^6^A abundance in cancer cells define the epigenetically heterogeneous (het) states where cells are genetically homogeneous (hom) in terms of targeting RTKs (KIT, FLT3, BCR/ABL). (ii) Cells with low FTO/high m^6^A are sensitive to TKI-induced killing. (iii) Cells carrying moderately high FTO/low m^6^A survive initially, and establish TKI resistance eventually when the FTO/m^6^A functions are further enhanced by chronic TKI exposure. (iv) Restoring m^6^A methylation by FTO inhibition resensitizes resistant cells to TKIs. Data in CCK-8 assays represent two independent experiments with 8 duplicates; Data are mean ± SD; *p *<* 0.05, **p *<* 0.01. See also Figure S8 and Table S3

To pursue the clinical relevance of FTO-induced m^6^A demethylation, we established nilotinib resistance in primary cells from both an AML and CML patient, respectively, by treating them with increasing concentrations of nilotinib till they could grow in 1 μM nilotinib-containing medium. Primary cells cultured in drug-free medium served as negative controls. While the colony assays did not show large differences regarding the average number, size and shape of colonies in resistant *vs* parental cells (not shown), the colony sizes within parental or resistant groups were not uniform (not shown). To avoid the possible interference from distinct colony sizes, we only selected similar clones (*n* = 5/group) for further characterization (Fig. 7a; Fig. S8). In agreement with the results from cell lines (see Fig. 1b), nilotinib dose-dependently decreased the proliferation rate of parental clones, but its inhibitory effects on resistant clones were minimal (Fig. 7b). However, treatment with rhein restored nilotinib sensitivity (Fig. 7c). Mechanistic studies revealed suppressed KIT- or BCR/ABL-STAT5 signaling (Fig. 7d, left), increased FTO levels (Fig. 7d, right), decreased mRNA m^6^A methylation (Fig. 7e) and upregulated *MERTK* and *BCL-2* (Fig. 7f) in resistant *vs* parental clones. In support of their sensitivity to rhein, exposure of resistant clones (*n* = 5) to rhein led to downregulation of *MERTK* and *BCL-2* (Fig. 7g).

To further examine the clinical implications of the FTO-m^6^A axis in patients, we obtained peripheral blood mononuclear cells (PBMCs) from 14 AML patients, who received 300 mg nilotinib twice daily on days 4–14 after induction and consolidation chemotherapy. The clinical characteristics of these patients are listed in Table S3. In agreement with the ex vivo results, nilotinib therapy resulted in *FTO* upregulation (3.1 ± 1.1 fold, day 7; 4.2 ± 1.3 fold, day 14; Fig. 7h). We then divided these patients into high or low *FTO* expression group, and subjected mRNA of high *FTO* group (*n* = 5) to m^6^A dotblotting for proof-of-principle studies. Compared to day 0, the levels of m^6^A methylation were notably decreased (Fig. 7i; Fig. S9). Thus, FTO-dependent m^6^A demethylation could have clinical implications in the management of TKI resistance, which warrants further conclusive studies involving a larger cohort of patients.

## Discussion

Our analysis has identified the FTO-m^6^A axis as a bona fide defense mechanism enabling leukemia cells to survive through TKI selection. This FTO-m^6^A axis appears to be intrinsic, pre-existing in naïve leukemia cell populations, and also be inducible in response to TKI treatment. On the one hand, the heterogeneous FTO-m^6^A axis allows a subpopulation of cells to rapidly prevail their m^6^A methylome in order to upregulate survival and proliferation genes so that they can withstand an initial onslaught of TKIs and continuously propagate in the absence of targeted kinases. On the other hand, the increased FTO-m^6^A function by TKIs further enhances expression of anti-apoptotic/survival genes, leading to establishment of resistant phenotypes. When m^6^A methylation is either genetically or pharmacologically restored, the resistant cells re-gain sensitivity to TKIs (Fig. 7J; Fig. S9).

It is well appreciated that the genetic heterogeneity in cancer cell clones influence the occurrence of acquired TKI resistance.^13,47^ However, little is known about which mechanisms define the heterogeneous states where cancer cell populations are genetically similar. Our current findings disclose that these genetically homogeneous leukemia cells, in terms of *BCR/ABL*, *KIT* or *FLT3* mutations, are epigenetically heterogeneous and can be distinguished by overexpression of *FTO* and reduction of m^6^A methylation. The fact that FTO-m^6^A activity is intrinsic and also inducible supports the idea that TKI resistant clones emerge from pre-existing drug-tolerant cells and *de novo* evolution.^13,47^ Mechanistically, as it is a dynamic and reversible action on mRNA,^14,26^ m^6^A methylation provides cells with yet another mode to greatly increase regulatory complexity and fine-tunes their transcriptome/proteome in the presence of TKIs. Unlike altered DNA methylation and histone modifications, which govern gene expression through chromatin remodeling, in drug resistance,^11,12^ FTO-dependent m^6^A hypomethylation modulates cell fate determinants, like *MERTK* and *BCL-2*,^10,37^ at least partially, through the enhanced RNA stability and protein translation.^48,49^ Notably, upregulation of proliferation/anti-apoptotic oncogenes bearing m^6^A sites are possibly determined by multiple mechanisms. In addition to m^6^A-regulated RNA stability and protein translation, transcriptional regulation through chromatin remodeling likely contributes to oncogenic overexpression and subsequent TKI resistance. It is important for future studies to address, to what extent, these transcriptional and posttranscriptional mechanisms regulate the development and maintenance of TKI resistance.

The levels of m^6^A methylation have been found to be regulated at different layers by methyltransferases,^17,18^ demethylases^19,20^ and m^6^A readers.^22,25,36^ However, this balance is shifted by FTO upregulation without notable changes in METTL3 expression in large naïve clones and resistant cells compared to their respective controls. Importantly, such FTO deregulation is required for leukemia cells to establish TKI resistance. First, intrinsic and ectopic *FTO* expression renders leukemia cells insensitive to TKI-induced lethality. Second, both genetic and pharmacologic FTO inactivation impairs resistant cell propagation and restores TKI sensitivity in vitro and in vivo. Third, although larger clones in naïve leukemia display an intrinsic TKI tolerance due to relatively high *FTO* expression, this TKI tolerance appears to be relatively moderate if normalized to the colony sizes. However, this “subtle” difference seems to be sufficient to initially help leukemia cell survive and proliferate under TKI-selective pressure. These cells that survive initially undergo evolution over time, with no genetic mutations occurring on the targeted RTKs. Instead, these cells develop hyperactive FTO leading to more pronounced m^6^A demethylation and subsequent higher TKI tolerance. Such inducible features of FTO-mediated m^6^A hypomethylation are further strengthened by the fact that TKI-resistant primary clones possess a more robust FTO-m^6^A pathway compared to parental clones, even when their clone sizes are similar, and that nilotinib therapy in AML patients increases *FTO* expression but deceases m^6^A methylation. Because FTO deregulation appears in naïve and resistant clones, this mechanism could be applied to both earlier stage leukemia, where FTO provides cells with initial defense against TKI killing, and also the advanced leukemia, where higher levels of FTO offer cancer cells more advantages to proliferate and progress. These proof of principle findings represent a major shift in understanding TKI resistance mechanisms, supporting FTO as an oncogene^28^ and a potentially druggable target to induce long-term response in TKI therapy. Further, the FTO/m^6^A axis could serve as a biomarker to predict response of the leukemia patients to the TKI treatment.

Finally, it’s worth noting that the m^6^A motifs/marks are largely conserved^14,50,51^ in given species and cell types,^14,27,35^ in line with our m^6^A-seq analysis showing that the distribution of m^6^A sites in transcriptomes is similar across nilotinib treatment. However, m^6^A methylation is dynamic, which m^6^A motifs are methylated and when it occurs largely depend on diverse conditions (e.g., heat shock, stages of disease/cell differentiation).^26,36^ Such dynamics could occur at multiple levels including, 1) changes in number of methylated transcripts (methylated at same loci), which result from the deregulated activity/expression of methyltransferases/demethylases (e.g., FTO); and/or 2) alterations in location of m^6^A motifs (resulting in loss or *de novo* peaks), which may be caused by a competition of FTO with other RNA binding proteins.^36^ These outcomes might reflect that FTO does not globally target all m^6^A-bearing mRNAs, but instead demethylates a subset (typically less than 10% of all methylated RNA) of mRNA.^28^ Our finding that the majority of differentially expressed genes did not show differential m^6^A peaks, that only FTO is consistently upregulated in resistant cells and that most m^6^A peaks are conservative across the diverse conditions, support the first hypothesis that FTO-dependent m^6^A abundance is one of the main factors regulating TKI resistance. However, the fact that a small number of genes display differential m^6^A peaks in nilotinibR cells, also supports the second level of m^6^A dynamics, which certainly merits further explorations in TKI resistance. Moreover, we and others^26^ demonstrated an inverse correlation between m^6^A methylation and gene expression, but we know many m^6^A-containing genes are downregulated when m^6^A becomes hypomethylated, as seen in TKI resistant cells. Thus, additional m^6^A-reading mechanisms must exist to stabilize methylated mRNAs. Moreover, numerous m^6^A-bearing genes, which are important to sustain resistance, had no or barely detectable changes in TKI resistant cells. As m^6^A participates in RNA splicing and other functions,^26,27,52^ it is possible that TKI selection links FTO-mediated m^6^A demethylation to isoform switching for these genes. The solutions to these puzzles will provide new insights into the m^6^A methylation in TKI resistance.

Collectively, our data support FTO-dependent m^6^A demethylation acting on mRNA, but not DNA and histones, as a hitherto unknown mechanism to allow selection of cells with survival advantages during chronic TKI exposure. These findings warrant further investigation of the dynamic m^6^A methylome in drug resistance and exploration of targeting the FTO-m^6^A axis as alternative approaches to counteract TKI resistance. Given the limited efficacy of available inhibitors for DNA methylation and histone modifications in cancer therapy, “RNA epitranscriptomic drugs”, including FTO inhibitors that revert m^6^A mRNA epigenomes from non-responsive cells to a drug responsive state, could be uniquely positioned to either prevent or override TKI resistance. As the functions of the FTO-m^6^A axis appear to be intrinsic and inducible, m^6^A modulation could be considered for use in a neo-adjuvant manner to prevent emergence of resistant clones, or to eradicate TKI resistance by impairing aberrant FTO demethylase activity that the resistant clones rely on to outcompete therapy-sensitive clones.

## Materials and methods

### Mice

Athymic nude mice (4–6 weeks old, male #490) were purchased from the Charles River. All animal procedures were performed according to NIH guidelines and approved by the Committee on Animal Care at the University of Minnesota. Mice were monitored daily for signs of deteriorating health as indicated by weight loss, slow movement or hunched posture etc.. All mice had free access to food and water throughout the study. To test the oncogenic potential of *FTO* gene, K562 parental cells were transfected with *FTO* expression or control vectors for 6 h, and 5.0 × 10^6^ transfected cells were subcutaneously injected into the bilateral flanks of the nude mice. For therapeutic experiments, K562 nilotinibR cells (1.5 × 10^6^) were injected subcutaneously into the bilateral flanks of the nude mice. To retain the resistance phenotypes, these mice received 1 mg/kg nilotinib twice a week for 4 weeks till the tumor approached ~10 mm^3^. Then the tumor-bearing mice were randomly divided into groups of 3 animals and received intraperitoneal injection of either 5 mg/kg nilotinib, 10 mg/kg rhein alone or their combination in polyethylene glycol 400 (PEG400, Sigma #1546445) and saline (ratio 15:38:47), three injections each week for 5 times in total. Mice were sacrificed on day 42 after the resistant cell inoculation and the tumors were harvested for H&E and IHC staining as well as molecular characterizations.

### AML and CML patient samples

The current study was approved by the Mayo Clinic Institutional Review Board and conducted in accordance with the Declaration of Helsinki. The diagnoses of AML and CML were made according to the criteria of World Health Organization. For ex vivo treatment, the primary cells from AML and CML patients with >80% blasts were cultured in RPMI-1640 plus 20% FBS. For in vivo nilotinib therapy, patients were enrolled if they were newly diagnosed with AML with *KIT* (CD117) expression of 20% or higher on myeloblasts by flow cytometry. *KIT* mutations were allowed if present. Nilotinib (300 mg) was given twice daily on days 4–14 of induction and consolidation. Cytarabine (100 mg/m^2^/day) continuous IV × 7 days plus daunorubicin (60 mg/m^2^) IV daily × 3 days were used for induction, while consolidation used standard cytarabine (3 gm/m^2^) twice daily on days 1, 3, 5 for a total of 4 cycles. PBMC (peripheral blood mononuclear cell) was prepared by Ficoll-Hypaque (GE Healthcare #71-7167-00) gradient centrifugation and directly used for molecular biological assays without further cell culture. All patients signed an informed consent document approved by the Institutional Review Board before entering any study.

### Cell lines and cell culture

Leukemia cell lines, K562, KU812, MV4-11 and Kasumi-1, were newly purchased from American Type Culture Collection with no further authentication or testing for mycoplasma. Cell lines were grown in RPMI-1640 (GE Healthcare #SH30027.01) supplemented with 20% (Kasumi-1) or 10% (K562, KU812 and MV4-11) fetal bovine serum (FBS, Gibco by Life Technologies^TM^ #16140-071) and Antibiotic-Antimycotic (Gibco by Life Technologies^TM^ #15240062) at 37 °C under 5% CO_2_. No cell line used in this paper is listed in the database of commonly misidentified cell lines maintained by ICLAC (International Cell Line Authentication Committee). The TKI resistant cells were cultured in RPMI-1640 (GE Healthcare #SH30027.01) containing 1 µM imatinib, nilotinib or PKC412, respectively.

### Plasmid design and construction

Human *FTO* expression vectors were constructed by inserting the *FTO* (ID: 79068) gene sequence into the pCDNA4 vector (ThermoFisher #V102020). The primers are:

*FTO* forward 5′-CTAGCTAGCATGAAGCGCAC-3′

*FTO* reverse 5′-CCGCTCGAGTCAGTGGTGGTGGTGGTGGTGCTAGGGTTTTGCTTCC-3′ The *YTHDF2* expression vector pcDNA3-flag-*YTHDF2* was purchased from Addgene (#52300). Three shRNAs against *FTO* (TRCN0000183897, V3LMM445071, V3LMM445074), *METTL3* (TRCN0000034715, TRCN0000034717, TRCN0000034718), *YTHDF2* (V2LHS_115145, V2LHS_115143, V3LHS_381614) and the negative control vectors (pLKO.1, pGIPZ) were obtained from BMGC RNAi (University of Minnesota).

### In vitro adaption of TKI resistant cells

Cell lines, K562, KU812, Kasumi-1 and MV4-11, and primary cells from leukemia patients were passaged with low concentration of imatinib, nilotinib or PKC412 (0.1 µM) and sequentially cultured in increasing concentrations of these TKIs (0.3, 1 µM) for 3 months. For resistance to second-line therapies, K562 and KU812 cells resistant to imatinib were sequentially treated with increasing concentrations of nilotinib starting at 0.1 µM. Cells cultured in parallel without drugs served as parental negative controls. Cells were considered resistant when they could routinely grow in medium containing 1 µM imatinib, nilotinib or PKC412, respectively.

### Transfection

Leukemia cells (1 × 10^6^/ml) were starved in fresh medium without Antibiotic-Antimycotic (Gibco by Life Technologies^TM^ #15240062) and FBS (Gibco by Life Technologies^TM^ #16140-071) for 2 h before transfection. The expression, shRNA and their respective control vectors were introduced into cells using Lipofectamine™ 2000 reagent (Life Technologies #11668-019) as previously reported.^53–56^ Notably, three shRNAs targeting different regions of each gene were utilized in 1:1:1 ratio (9 μg in total) in 10 × 10^6^ cells. The shRNA-transfected cells were selected in RPMI-1640 containing 1 μg/ml puromycin (ThermoFisher #BP2956) for 48 h before further investigations.

### m^6^A dotblotting

The total RNA was extracted using miRNeasy Mini Kit (Qiaqen #217004) and the mRNA was purified by GenElute™ mRNA Miniprep Kit (Sigma #MRN70). In control experiments (K562 and Kasumi-1 parental or nilotinibR cells), the rRNA was further cleaned using the RiboMinus Transcriptome Isolation Kit (Invitrogen #K155002). About 2 μg mRNA was denatured and subjected to dotblotting using anti-m^6^A antibody (Synaptic Systems #202003) as previously described.^26,35^ The RNA spotted membrane was stained with 0.02% methylene blue (Sigma #1808) in 0.5 M sodium acetate (pH 5.0) for loading control.

### Western blotting

The whole cellular lysates were prepared by harvesting the cells in 1 × cell lysis buffer (20 mM HEPES (pH 7.0), 150 mM NaCl and 0.1% NP40) supplemented with 1 mM phenylmethane sulfonyl fluoride (PMSF, Sigma #10837091001), 1 × Phosphatase Inhibitor Cocktail 2 and 3 (Sigma #P5726, P0044), and 1 × protease inhibitors (protease inhibitor cocktail set III, Calbiochem-Novabiochem #539134). The proteins were resolved by sodium dodecyl sulfate (SDS)–polyacrylamide gel electrophoresis, transferred onto PVDF membranes (GE Healthcare #10600023), blocked by 5% non-fat milk followed by probing with anti-STAT5 (1:1000, Cell Signaling #9363), anti-p-STAT5 (1:1000, Cell Signaling #9351), anti-KIT (1:500, Cell Signaling #3074), anti-p-KIT (1:500, Cell Signaling #3391), anti-FLT3 (1:500, Cell Signaling #3462), anti-p-FLT3 (1:500, Cell Signaling #3464), anti-BCR (1:500, Cell Signaling #3902), anti-p-ABL (1:500, Cell Signaling #2865), anti-β-Actin (1:1000, Santa Cruz #sc-47778), anti-MERTK (1:1000, Santa Cruz #sc-365499), anti-FTO (1:2000, AdipoGen #AG-20A-0064), anti-METTL3 (1:2000, Proteintech #15073-1-AP), anti-ALKBH5 (1:500, Abcam #ab69325), anti-METTL14 (1:1000, Abcam #ab98166), anti-WTAP (1:2000, Abcam #ab195380), and anti-BCL-2 (1:500, EMD Millipore #05-729) antibodies. The secondary antibodies are: horse anti-mouse IgG, HRP-linked antibody (Cell Signaling #7076), goat anti-rabbit IgG, HRP-linked antibody (Cell Signaling #7074), rabbit anti-goat IgG HRP-linked antibody (Invitrogen #31402).

### RNA isolation, cDNA preparation and quantitative PCR (qPCR)

According to the Kit instructions, the total RNA was isolated using miRNeasy Kit (Qiaqen #217004), and complementary DNA (cDNA) synthesis was performed using SuperScript® III First-Strand Synthesis System (Invitrogen #18080-051). The expression of target genes was assessed by SYB Green qPCR (Applied Biosystems #4309155). The levels of *GAPDH* were used as normalization and the expression of the targets was analyzed using the ΔCT approach. The primers used are:

*E2F2* forward 5′-AGTAGAGTTGGAGATTCA-3′

*E2F2* reverse 5′-CTCTGGTCTATTCTAACAC-3′

*COL18A1* forward 5′-AAGATTCCAGAAGTGAAGAAGT-3′

*COL18A1* reverse 5′-ATCTGAGCCAGGAAGTGT-3′

*CTSB* forward 5′-ATCCTCGTCAACCTTCTC-3′

*CTSB* reverse 5′-TCAGTCACAACAACAACAA-3′

*CITED2* forward 5′-CCTCCCTTATGTAGTTGAAAT-3′

*CITED2* reverse 5′-ATCCACAAGATTAAGCAGTT-3′

*MERTK* forward 5′-CATTCACAGAGGAGGATT-3′

*MERTK* reverse 5′-GCTATGTAAGGTAAGTTCAAT-3′

*ID2* forward 5′-CACAACAACAACAACAAC-3′

*ID2* reverse 5′-CACAGTCCAAGTAAGAGA-3′

*MTHFR* forward 5′-TTGGCACAGTTGTCTATTCT-3′

*MTHFR* reverse 5′-CAAGCACAAGGGTTCAGA-3′

*ULK1* forward 5′-GTCACACGCCACATAACAG-3′

*ULK1* reverse 5′-TCTTCTAAGTCCAAGCACAGT-3′

*SIPA1* forward 5′-AAGGCTTTGAGAGTTACC-3′

*SIPA1* reverse 5′-TCCTGGTATGTGGTGTAG-3′

*EME1* forward 5′-CTGACTGTAATGAAGAGA-3′

*EME1* reverse 5′-TAATAAGACCCAAGAAAGA-3′

*SLC29A1* forward 5′-GTCTCTGTGTATGTGTCT-3′

*SLC29A1* reverse 5′-AATGGAGTATATCAGGTCAA-3′

*FTO* forward 5′-TTCACCAGCATAGTATAGTT-3′

*FTO* reverse 5′-AGTCTCCAATGTCATCAG-3′

*BCL-2* forward 5′-ACATCCTATCAACAACAA-3′

*BCL-2* reverse 5′-GTATCTACACTACAGTCTTA-3′

*s-MERTK* forward 5′-ATTCTTCTGCTGTAGGAG-3′

*s-MERTK* reverse 5′-GCTTACTTAACAACATTCATC-3′

*s-BCL-2* forward 5′-TTAAGAGGTGGCTGATAT-3′

*s-BCL-2* reverse 5′-TTAATGGCAATGTGACTT-3′

*EMILIN3* forward 5′-TTGTATCTGAACTAAGGA-3′

*EMILIN3* reverse 5′-AAAGGGAATAAAGAGAAC-3′

*SSH2* forward 5′-TTGCTCAGTAGTCTCCTT-3′

*SSH2* reverse 5′-TCCTCACATTGGTTCATAC-3′

*GAPDH* forward 5′-GAGTCAACGGATTTGGTCGT-3′

*GAPDH* reverse 5′-GACAAGCTTCCCGTTCTCAG-3′

### Gene expression microarray

Total RNA was extracted from K562 nilotinibR and parental cells using RNeasy Mini Kit (Qiagen #74104) and subjected to gene expression analysis using HumanHT-12v4 Expression BeadChip Kit (Illumina). Briefly, total RNA samples were processed using the Illumina TotalPrep-96 RNA Amplification Kit for High-Throughput RNA Amplification for Array Analysis (Part# 4393543). About 300 ng of total RNA were first processed in a reverse transcription reaction. The cDNA then underwent second strand synthesis and cleanup to become a template for in vitro transcription (IVT). The IVT generated biotinylated, antisense RNA copies of each mRNA in a sample. After normalization in concentration, samples were loaded onto the appropriate Illumina Beadchip. Hybridization was proceeded overnight. Beadchips were then washed, stained, and scanned using the Illumina iScan Beadarray Reader. Data were then processed and basic quality metrics were checked using Illumina Genome Studio. Robust multichip average (RMA) 24 within RMA Express 1.0 was used for data normalization. BRB-ArrayTools (http://linus.nci.nih.gov/BRB-ArrayTools.html) was used for the group comparisons with a 2-sample *t* test for each gene and a univariate test significance of 0.001. For a gene to be considered significantly up- or down-regulated, the *p* value of the univariate test should be <0.05, the fold difference is ≥1.5. The signaling pathway was analyzed using the DAVID bioinformatics resources (Version 6.7).

### mRNA degradation and protein translation assays

The transfected and drug-treated cells were treated with actinomycin-D (Cayman Chemical #11421) at a final concentration of 5 µg/ml for the indicated time points. The total RNA was purified by TRIzol^®^ RNA isolation reagent (Invitrogen #15596018). The value recorded was the percentage of mRNA remaining compared with the amount before the addition of actinomycin-D (Cayman Chemical #11421) after normalization to the levels of *GAPDH*. For protein translation assays, cells were treated with puromycin (ThermoFisher #BP2956) at a final concentration of 200 ng/ml followed by incubation for indicated time points. The cells were lysed for the Western blotting and the protein expression was normalized to β-actin (1:1000, Santa Cruz #sc-47778).

### m^6^A immunoprecipitation (IP)

The RNAs were chemically fragmented into 100-nucleotide-long fragments by 5 min incubation at 94 °C in fragmentation buffer (10 mM ZnCl_2_, 10 mM Tris-HCl, pH 7.0). The fragmentation reaction was stopped with 0.05 M EDTA followed by standard ethanol precipitation. The fragmented RNAs were resuspended in H_2_O at 1 mg/ml concentration and incubated for 2 h at 4 °C with 5 µl anti-m^6^A antibody (Synaptic Systems #202003) in IPP buffer (150 mM NaCl, 0.1% NP-40, 10 mM Tris-HCl, pH 7.4). The mixture was then immunoprecipitated by incubation with Dynabeads™ Protein G (ThermoFisher #10004D) at 4 °C for additional 2 h. After extensive washing by IPP buffer, bound RNAs were eluted from the beads with 0.5 mg/ml *N*^*6*^-methyladenosine (Selleck Chemicals #S3190) in IPP buffer and ethanol precipitated for further investigations.

### m^6^A sequencing and data processing

The mRNA was isolated from total RNA using Dynabeads mRNA DIRECT Purification Kit (ThermoFisher #61011), adjusted to 15 ng/µl in 100 µl and fragmented using Bioruptor ultrasonicator (Diagenode) with 30 s on/off for 30 cycles. The m^6^A-immunoprecipitation (m^6^A-IP) and library preparation were performed according to the published protocol.^57^ Sequencing was carried out on Illumina HiSeq 4000 following the manufacturer’s instructions. All data were sequenced with single-end 50-bp read length. For both m^6^A-IP and input, reads were aligned to reference genome hg38 using Tophat v2.1.1^58^ with parameter -g1. Refseq gene annotation was downloaded from USCS. The m^6^A peak calling was performed using MeTPeak^59^ with parameters FRAGMENT_LENGTH = 200. Differential m^6^A peak calling was called using MetDiff^60^ with parameters FRAGMENT_LENGTH = 200. The metagene plot was generated using Guitar.^61^ Homer v4.9.1^62^ was used to search for enriched motif in m^6^A peak region. The parameter used in Homer was findMotifsGenome.pl-bg longest_isoform.bed-rna-len 5, 6, 7 where longest isoform of human transcriptome was used as background sequence for motif discovery.

### Peak reads count analysis for m^6^A sequencing

Peaks were called for each of the three groups by using MeTPeak R package as described previously.^59^ The union of peaks in three groups was obtained using custom script. Specifically, if there was a peak called in any or multiple of the groups, it would be included in the union peaks. Then the number of reads falling into each peak region of the union peaks was counted for input library and m^6^A-IP library. To calculate the total number of reads falling into peak region in input and IP sample, we summed the reads across genes for each sample and normalized them to total mRNA reads.

To calculate the fold enrichment of each peak, we used two alternative widely used formulas. In the first formula, fold enrichment was calculated by a/b, where (a) is the number of reads falling into peak region of IP sample; (b) is the number of reads falling into peak region of input sample. An alternative formula calculated fold enrichment by (a/c)/(b/d), where (c) is the median reads count of 100 bp sliding windows on the peak-containing-gene for the IP sample and (d) is the median reads count of the same sliding windows for input sample. To compare the overall fold enrichment across samples, we took the mean (and median) of the peak fold enrichment across peaks for each sample. For two replicates of each group, we took the mean fold enrichment for each peak. Then fold change of enrichment across groups (e.g. from Parental to NilotinibR) was calculated by log_2_(nilo)-log_2_(parental) for each peak. The distribution of log_2_ fold change was plotted by histogram. The coverage plot was generated using R package m^6^Amonter https://github.com/scottzijiezhang/m6Amonster.

### Hematoxylin and eosin (H&E) and Immunohistochemistry (IHC) staining

Tumors and tissues collected from the animal studies were immediately fixed in 10% neutral buffered formalin. The paraffin-embedded samples were cut to 5 μm thick and stained with H&E (Sigma #H9627, HT100180). For IHC staining, slides were heated in a dry oven at 60 °C for 30 min, deparaffinized in Histo-Clear for 10 min, rehydrated through graded ethanol (100, 95 and 70%, respectively, for 5 min) and rinsed in deionized water. All section slides were then microwaved in 10 mM citric acid buffer (pH 6.0) at 70% power for 10 min to unmask antigens. Endogenous peroxidase was quenched using 3% hydrogen peroxide for 20 min followed by rinsing with PBS. Nonspecific binding was blocked with 10% horse serum for 40 min, then with avidin and biotin (Vector Laboratories #SP-2001) for 15 min each. Primary antibodies against Ki-67 (1:1000, Abcam #ab15580) and FTO (1:2000, AdipoGen #AG-20A-0064) were used for incubation at room temperature for 1 h, respectively. For detection of primary antibodies, the Vectastain Elite ABC-peroxidase Universal Kit (Vector Laboratories, #PK-6200) was used. Samples were developed with 3, 3′-diaminobenzidine (Vector Laboratories #SK-4105), counterstained with hematoxylin #HK100-9K, and mounted. Stained slides were viewed and photographed with a Leica microscope mounted with a high-resolution spot camera, which is interfaced with a computer loaded with Image-Pro Plus software.

### Clonogenic assays

Colony-forming assays were performed in MethoCult^®^ medium (Stem Cell Technologies #03434) as previously reported.^53–56^ Briefly, the transfected or drug-treated cells were suspended in 0.3 mL of IMDM medium (Stem Cell Technologies #36150), mixed with MethoCult^®^ medium and then dispensed into 35 mm dishes. Colonies were counted in 9–14 days.

### Cell proliferation and apoptosis assays

Cell proliferation assays were performed using Cell Counting Kit-8 (CCK-8, Dojindo Molecular Technologies #CK04-11) as previously reported.^54,56,63^ Briefly, the parental and resistant cells (1.5 × 10^4^) in RPMI-1640 medium (100 µl) were dispensed into 96-well flat-bottomed microplates and drugs were added after 24 h of incubation. The cells were cultured for another 24 or 48 h, and CCK-8 (10 µl) was added to each well. The microplates were incubated at 37 °C for additional 2~4 h. Absorbance was read at 450 nm using a microplate reader and the results were expressed as a ratio of the treated over untreated cells (as 100%). Four wells were sampled per each experimental group in a given experiment. Averages are reported ± SD.

Cell apoptosis assays were performed using Annexin V-PI Apoptosis Detection Kit I (BD Pharmingen^TM^ #556547) according to the manufacturer’s instruction, and followed by flow cytometry analysis. The EdU incorporation assays were performed using Click-iT™ EdU Alexa Fluor™ 647 Flow Cytometry Assay Kit (ThermoFisher #C10419) according to the manufacturer’s instruction.

### Statistical analysis

The statistical analysis was performed using the Student’s *t* test. All analyses were performed using the GraphPad Prism 5 Software. In CCK-8 assays, the IC_50_ values were calculated using the GraphPad Prism 5 Software and showed as the mean of two independent experiments with 8 repeats. The cell proliferation curves were generated automatically by GraphPad Prism 5 Software on the basis of these IC_50_ values. *p* *<* 0.05 was considered statistically significant. All *p* values were two-tailed. No blinding or randomization was used. No samples or animals were excluded from analysis. All criteria were pre-established. No statistical method was used to predetermine sample size and the sample size for all experiments (in vitro and in vivo) was not chosen with consideration of adequate power to detect a pre-specified effect size. Variations were compatible between groups. In vitro experiments, such as qPCR, Western blotting, cell proliferation assays, dotblotting, clonogenic assays etc. were routinely repeated three times unless indicated otherwise in Figure legends or main text. For every Figure, the statistical tests were justified as appropriate.

### Data availability

Gene expression microarray data have been deposited in Gene Expression Omnibus (GEO) with the accession code GSE80481.

## Electronic supplementary material

Supplementary information, Figure S1

Supplementary information, Figure S2

Supplementary information, Figure S3

Supplementary information,  Figure S4

Supplementary information, Figure S5

Supplementary information, Figure S6

Supplementary information, Figure S7

Supplementary information, Figure S8

Supplementary information, Figure S9

Supplementary information, Table S1

Supplementary information, Table S2

Supplementary information, Table S3
