# Supplementary material for: A dynamic N6-methyladenosine methylome regulates intrinsic and acquired resistance to tyrosine kinase inhibitors
Source: Cell Res. 2018 Oct 8;28(11):1062–76. doi: 10.1038/s41422-018-0097-4 (PMC6218444; doi:10.1038/s41422-018-0097-4)
Supplement: Supplementary file 1 — Supplementary information, Figure S1 [file 41422_2018_97_MOESM1_ESM.pdf]

Figure S1

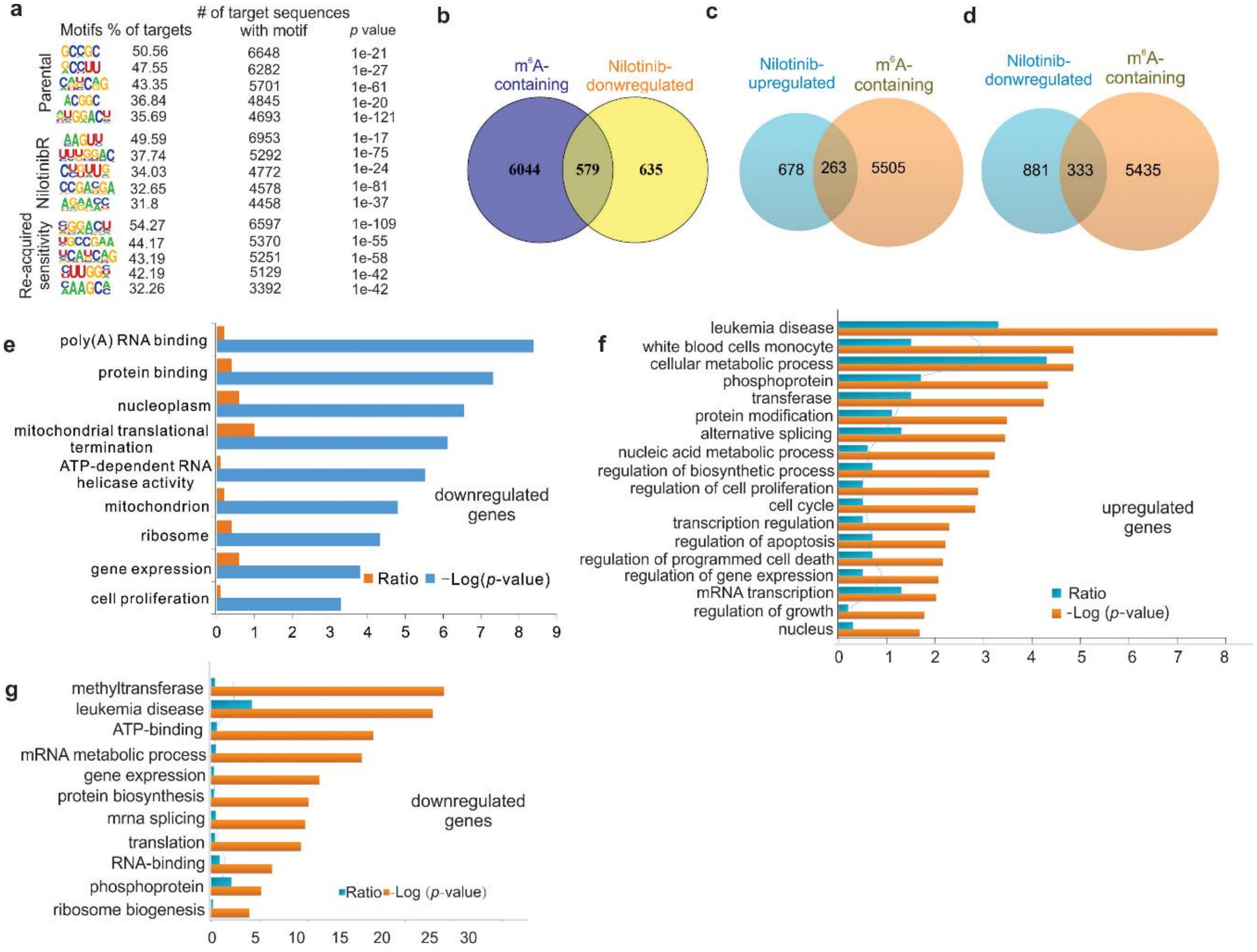

Figure S1. The global view of genes/pathways involving m^{6}A sites in K562 cells. **a** Target sequence motifs identified by HOMER motif analysis in K562 parental, nilotinib resistant cells and the cells re-sensitive to nilotinib. **b-d** Venn diagram illustrating the number of overlapped genes between differentially expressed genes and those with potential m^{6}A sites in parental K562 (**b**) or HEK293 (**c**,**d**) cells. **e-g** Enrichment scores for GO categories in overlapped genes. The –log (*p* value) axis indicates the statistical significance of the functions to the datasets.
