# Supplementary material for: A dynamic N6-methyladenosine methylome regulates intrinsic and acquired resistance to tyrosine kinase inhibitors
Source: Cell Res. 2018 Oct 8;28(11):1062–76. doi: 10.1038/s41422-018-0097-4 (PMC6218444; doi:10.1038/s41422-018-0097-4)
Supplement: Supplementary file 2 — Supplementary information, Figure S2 [file 41422_2018_97_MOESM2_ESM.pdf]

Figure S2

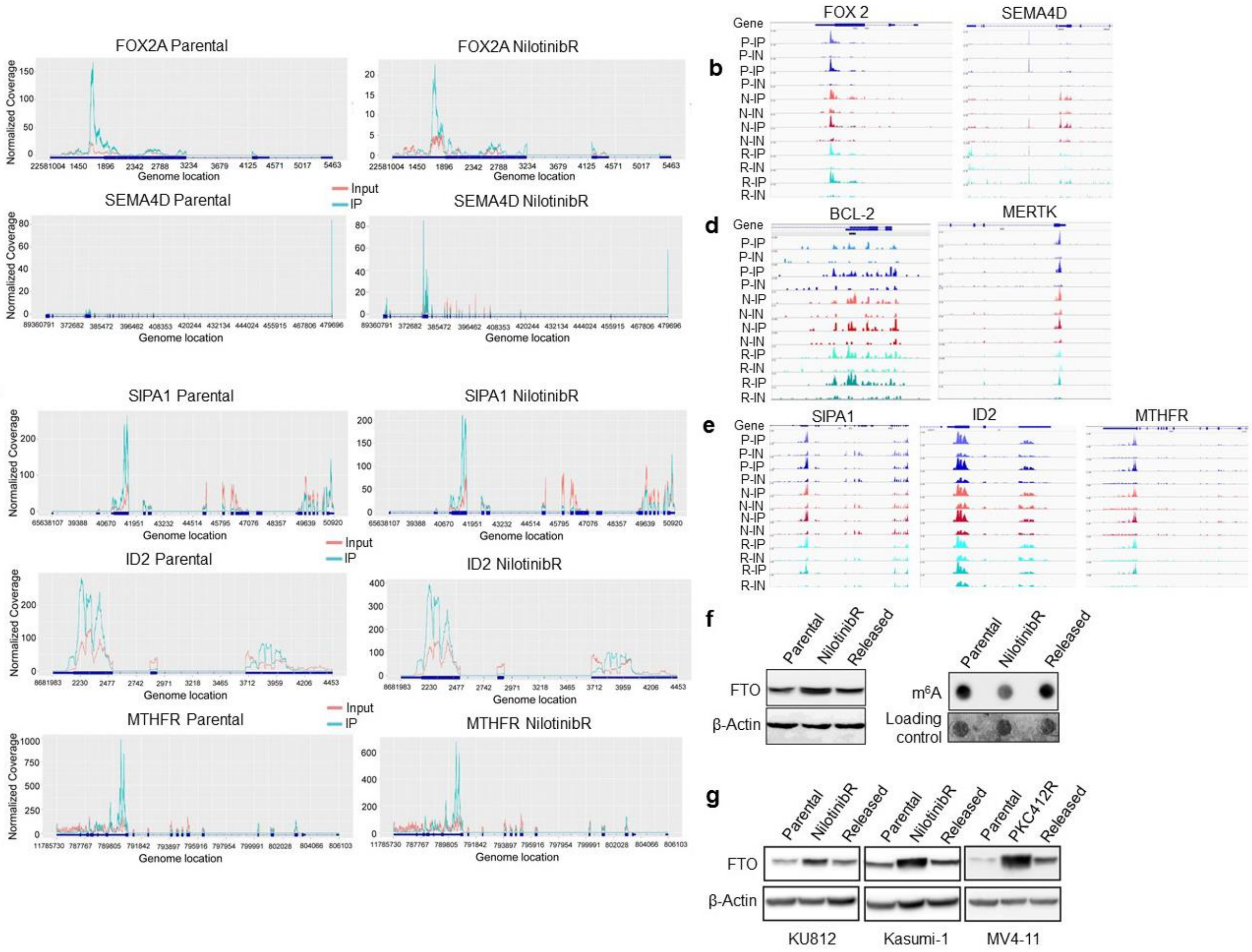

Figure S2. Characterization of m^{6}A changes and FTO expression in different cell types. **a** Coverage plots of m^{6}A IP and input reads (without smoothing the curve). Examples of differentially methylated transcripts harboring m^{6}A peaks exposing to nilotinib are shown. **b** IGV tracks displaying examples of sequencing read clusters from two m^{6}A-seq replicates are shown. **c** Coverage plots of m^{6}A IP and input reads (without smoothing the curve). **d**,**e** IGV tracks displaying examples of sequencing read clusters from two m^{6}A-seq replicates are shown. **f** Western blotting (left) and dotblotting (right) of K562 parental, nilotinibR and released cells. **g** Western blot for FTO expression. The resistant cells were cultured in drug-containing or drug-free medium for 15 days. The parental cells were used as controls.

IP, immunoprecipitation; IN, input; P, parental; N (Nilo), nilotinibR; R, reacquired sensitivity (released); In **b**, **d** and **e**, IP = blue, Input = red.
