# Supplementary material for: A dynamic N6-methyladenosine methylome regulates intrinsic and acquired resistance to tyrosine kinase inhibitors
Source: Cell Res. 2018 Oct 8;28(11):1062–76. doi: 10.1038/s41422-018-0097-4 (PMC6218444; doi:10.1038/s41422-018-0097-4)
Supplement: Supplementary file 3 — Supplementary information, Figure S3 [file 41422_2018_97_MOESM3_ESM.pdf]

Figure S3

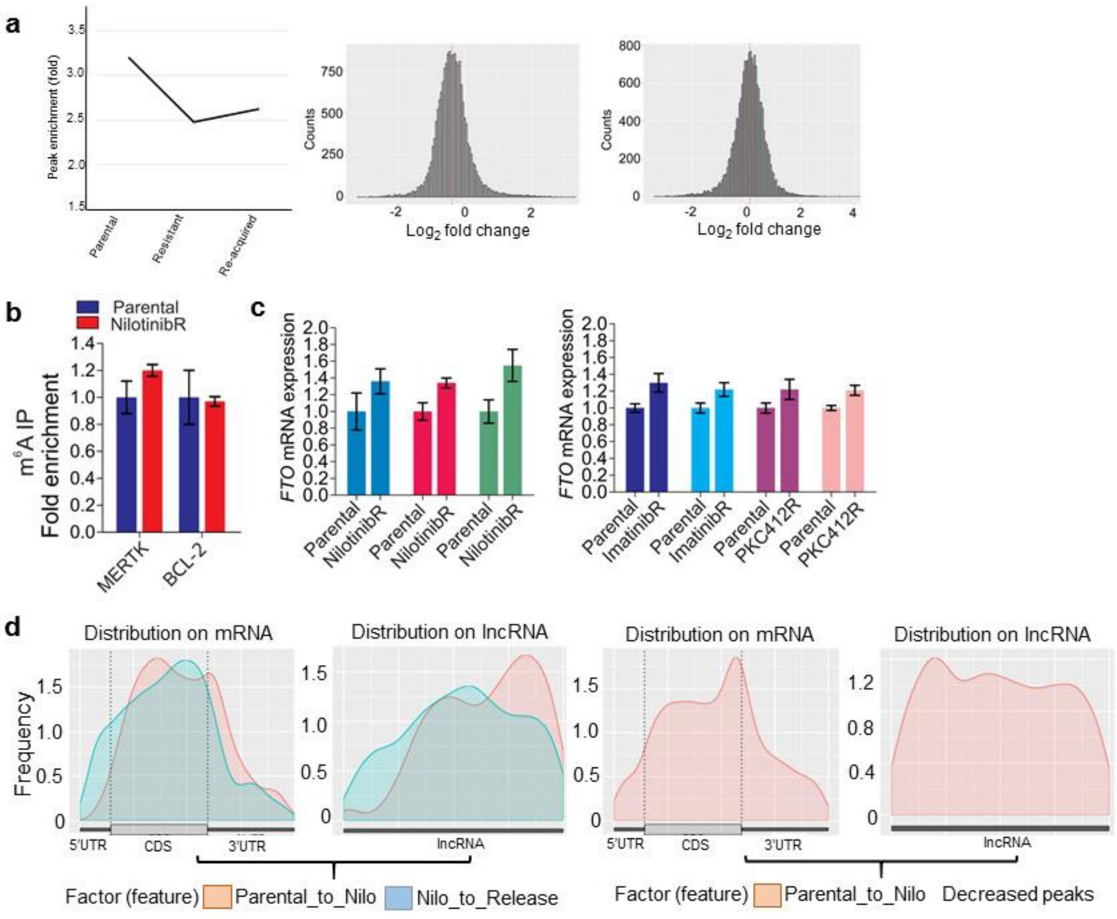

Figure S3. Characterization of *FTO* expression and m^6A peaks. **a** Analysis of m^6A peak read accounts. Left, enrichment fold difference was calculated by the total read counts across all peaks in input and IP, and then normalized by total mappable reads for each library; middle and right, plot distribution of $\log_2$ change of enrichment across m^6A peaks from parental to resistant (middle) or from resistant to reacquired sensitivity to nilotinib (right). The average $\log_2$-transformed normalized signal for the duplicated m^6A-seq was used to generate a histogram of read counts values. **b** The eluted mRNA from anti-m^6A immunoprecipitates in parental and resistant cells was subjected to cDNA synthesis followed by qPCR for gene expression using primers outside m^6A motifs. **c** qPCR of parental and resistant cells for *FTO* expression. **d** Metagene plots of differential

m^6A peaks across groups. Left, differential peaks (both increased and decreased) of Parental-to-Nilo comparison and Nilo-to-Release comparison; right, peaks showing the decreased enrichment from Parental-to-Nilo comparison.

Nilo, NilotinibR; Release, reacquired sensitivity.
