# Supplementary material for: A dynamic N6-methyladenosine methylome regulates intrinsic and acquired resistance to tyrosine kinase inhibitors
Source: Cell Res. 2018 Oct 8;28(11):1062–76. doi: 10.1038/s41422-018-0097-4 (PMC6218444; doi:10.1038/s41422-018-0097-4)
Supplement: Supplementary file 4 — Supplementary information, Figure S4 [file 41422_2018_97_MOESM4_ESM.pdf]

Figure S4

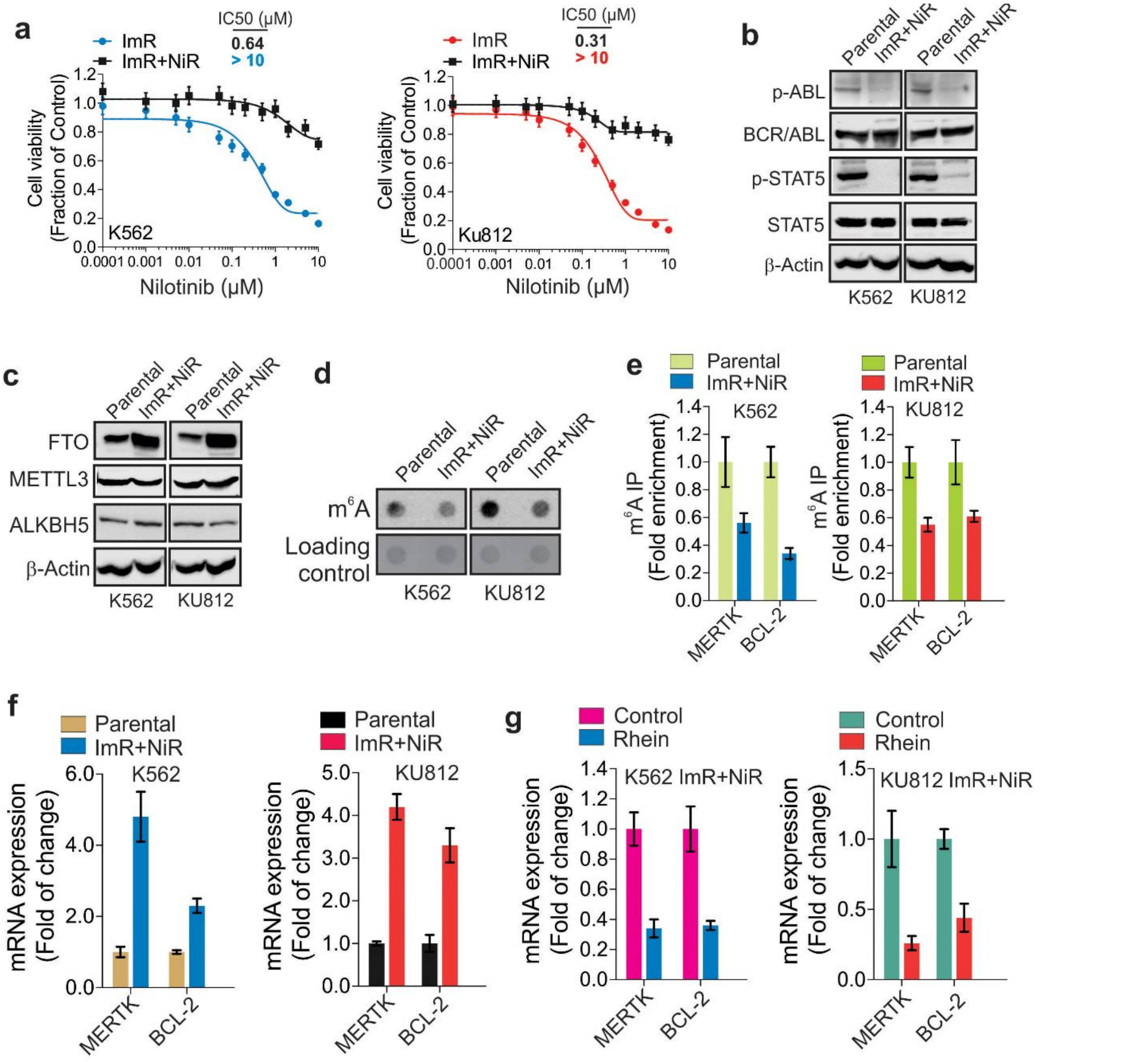

Figure S4. FTO upregulation and m^{6}A demethylation occur in second-line resistant cells. **a** CCK-8 assays in first- (ImR) and second- (ImR+NiR) line K562 and KU812 resistant cells treated with nilotinib for 72 hours. The data represent two independent experiments with 8 repeats in total. **b**,**c** Western blotting of parental and ImR+NiR cells. **d** Dotblotting of parental and ImR+NiR cells. **e** The mRNA from parental and ImR+NiR cells was subjected to immunoprecipitation (IP) by anti-m^{6}A. The eluted RNA was subjected to cDNA synthesis followed by qPCR for gene expression.

f,**g** qPCR of parental *vs* ImR+NiR cells (**f**) or ImR+NiR cells treated with 25 μM rhein for 48 hours (**g**).

In **b-g**, data represent three independent experiments.
