# Supplementary material for: A dynamic N6-methyladenosine methylome regulates intrinsic and acquired resistance to tyrosine kinase inhibitors
Source: Cell Res. 2018 Oct 8;28(11):1062–76. doi: 10.1038/s41422-018-0097-4 (PMC6218444; doi:10.1038/s41422-018-0097-4)
Supplement: Supplementary file 5 — Supplementary information, Figure S5 [file 41422_2018_97_MOESM5_ESM.pdf]

Figure S5

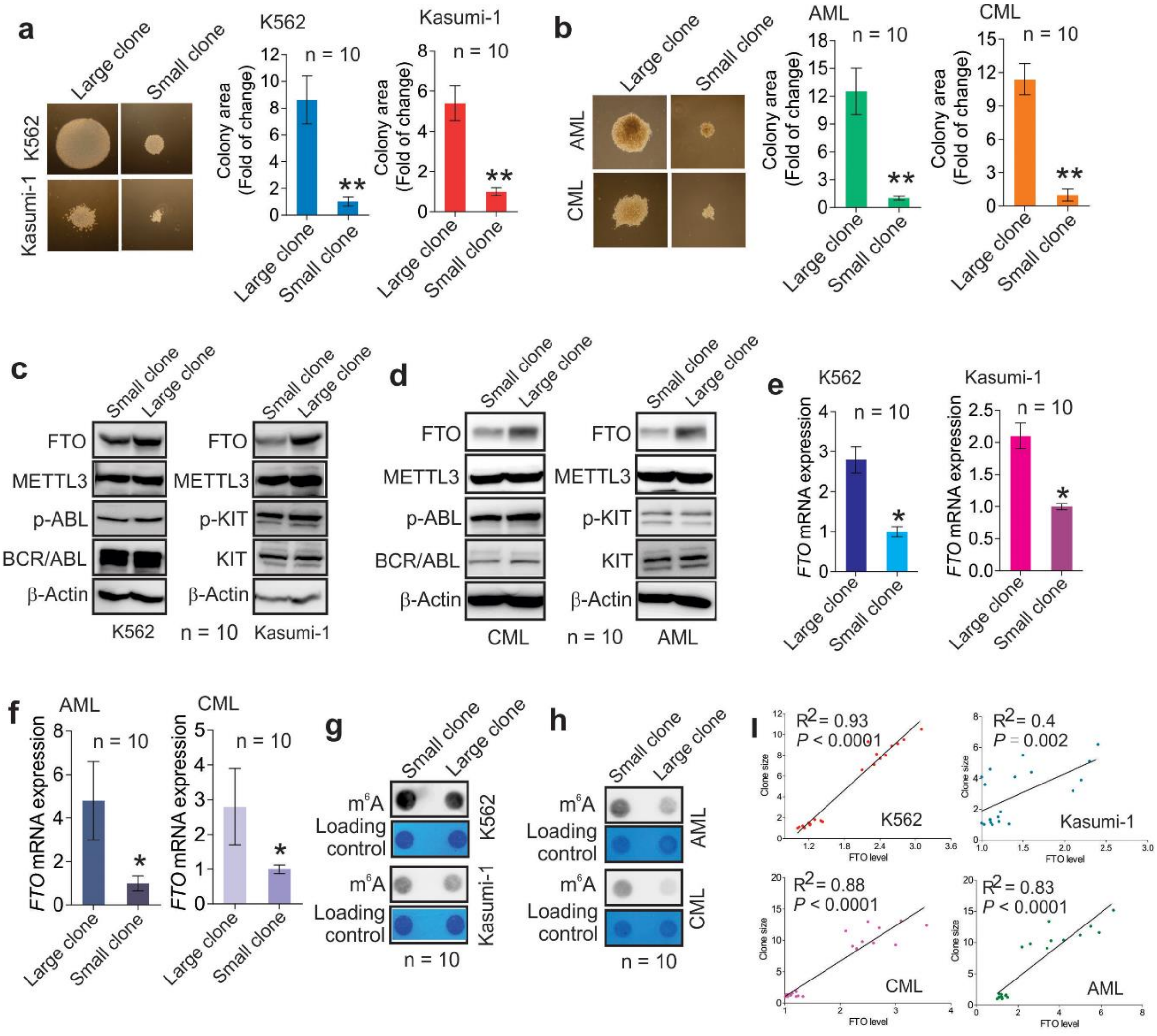

Figure S5. The FTO-m^{6}A axis reflects the heterogeneous nature of leukemia cell populations. The leukemia cell lines (K562, Kasumi-1) and patient primary cells (CML, AML) were subjected to colony-forming assays and the single colonies (n = 10/cell type/group) were selected for further investigations. **a**,**b** Left, representative images of colonies; graph is the quantification of colony sizes. **c**,**d** The protein lysates of 10 colonies were pooled and subjected to Western blotting. **e**,**f** qPCR for *FTO* expression in large and small colonies. **g**,**h** The mRNA from 10 colonies were pooled and subjected to dotblotting. Data are mean ±SD; *$p < 0.05$, **$p < 0.01$. **i** Scatter plot

showing the correlation of *FTO* expression and colony sizes reported in (**a**) and (**b**). R: Pearson correlation coefficients; R^2: means 'the goodness of fit'. Statistical significance was calculated by Pearson correlation coefficients.
