# Supplementary material for: A dynamic N6-methyladenosine methylome regulates intrinsic and acquired resistance to tyrosine kinase inhibitors
Source: Cell Res. 2018 Oct 8;28(11):1062–76. doi: 10.1038/s41422-018-0097-4 (PMC6218444; doi:10.1038/s41422-018-0097-4)
Supplement: Supplementary file 6 — Supplementary information, Figure S6 [file 41422_2018_97_MOESM6_ESM.pdf]

Figure S6

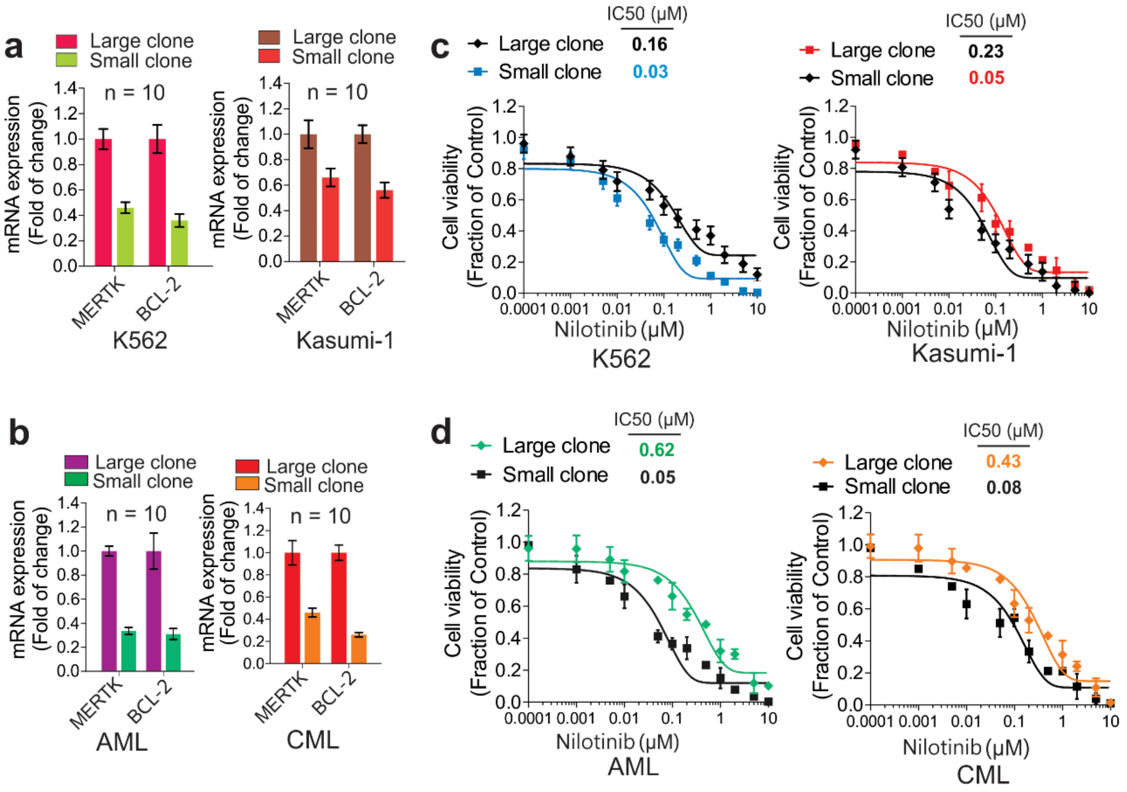

Figure S6. The lager clones have less sensitivity to nilotinib. The leukemia cell lines (K562, Kasumi-1) and patient primary cells (CML, AML) were subjected to colony-forming assays and the single colonies (n = 10/cell type/group) were selected for further investigations. **a**,**b** qPCR for the expression of the representative drug resistant genes in large and small colonies. **c**,**d** CCK-8 assays in large and small colonies treated with nilotinib for 72 hours. Data represent two independent experiments for three representative colonies with 12 repeats in total.
