# Supplementary material for: A dynamic N6-methyladenosine methylome regulates intrinsic and acquired resistance to tyrosine kinase inhibitors
Source: Cell Res. 2018 Oct 8;28(11):1062–76. doi: 10.1038/s41422-018-0097-4 (PMC6218444; doi:10.1038/s41422-018-0097-4)
Supplement: Supplementary file 7 — Supplementary information, Figure S7 [file 41422_2018_97_MOESM7_ESM.pdf]

Figure S7

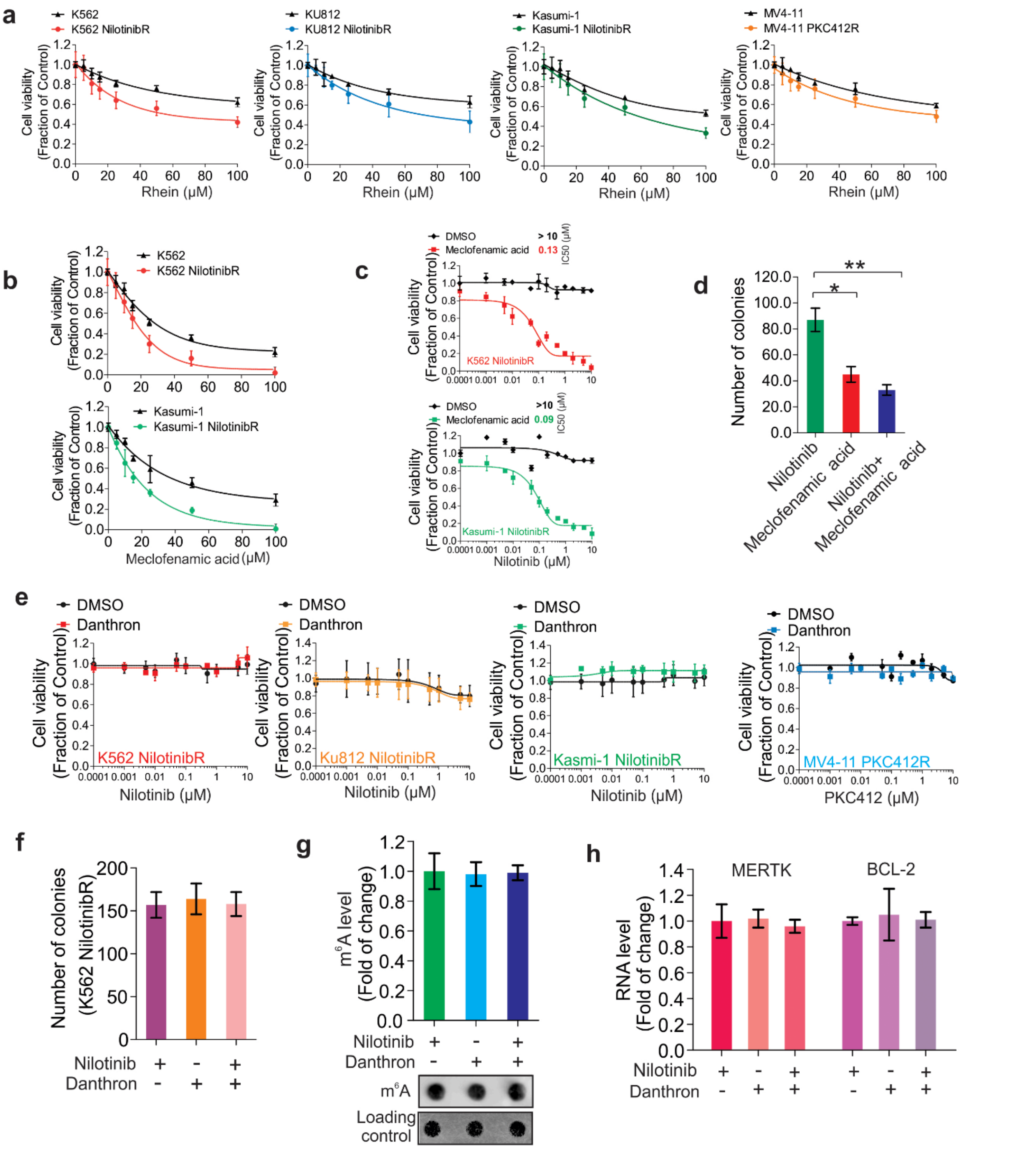

Figure S7. Resistant cells are more sensitive to FTO inhibitor-induced cell growth arrest. **a** CCK-8 assays in parental and resistant K562, KU812, Kasumi-1 and MV4-11 cells treated with various doses of rhein for 72 hours. **b** CCK-8 assays in parental and resistant K562 or Kasumi-1 cells

treated with various doses of meclofenamic acid for 72 hours. **c** CCK-8 assays in K562 or Kasumi-1 nilotinibR cells treated with 50 μM meclofenamic acid for 6 hours and then co-treated for 72 hours with meclofenamic acid plus varying concentrations of nilotinib. The effect of drug combination was normalized to DMSO only. **d** Colony-forming assays in K562 nilotinibR cells treated with 1 μM nilotinib or/and 50 μM meclofenamic acid. **e** CCK-8 assays in resistant cells treated with 10 μM danthron for 6 hours and then co-treated for 72 hours with danthron plus varying concentrations of nilotinib. **f-h** Resistant K562 cells were treated with either nilotinib, danthron alone or both for 48 hours and subjected to colony assays (**f**), Dotblotting (**g**) or qPCR (**h**).

Data in CCK-8 assays represent two independent experiments with 8 repeats in total; Data in Dotblotting and qPCR represent three independent experiments; Data in colony assays represent two independent experiments with four repeats in total; Data are mean ±SD.
