# Supplementary material for: A dynamic N6-methyladenosine methylome regulates intrinsic and acquired resistance to tyrosine kinase inhibitors
Source: Cell Res. 2018 Oct 8;28(11):1062–76. doi: 10.1038/s41422-018-0097-4 (PMC6218444; doi:10.1038/s41422-018-0097-4)
Supplement: Supplementary file 8 — Supplementary information, Figure S8 [file 41422_2018_97_MOESM8_ESM.pdf]

Figure S8

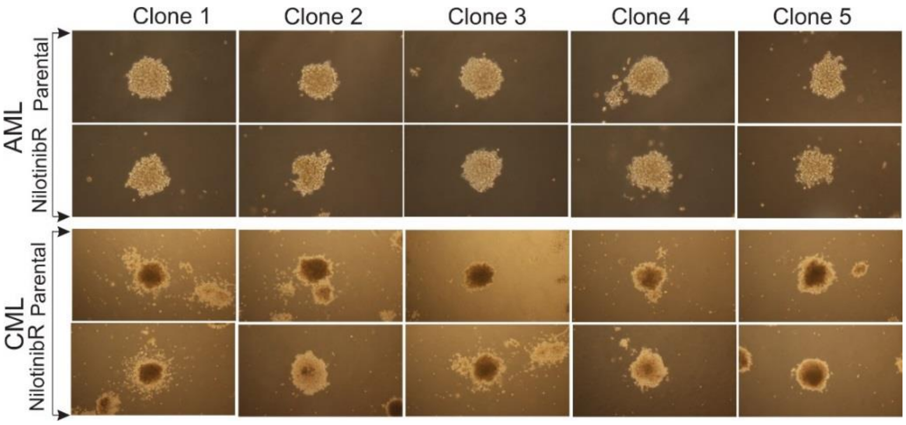

Figure S8. External view of colonies. The primary cells from AML and CML patients were passaged with low concentration of nilotinib (0.1 μM) and sequentially cultured in increasing concentrations of nilotinib (0.3, 1 μM) for 3 months.
