# Supplementary material for: A dynamic N6-methyladenosine methylome regulates intrinsic and acquired resistance to tyrosine kinase inhibitors
Source: Cell Res. 2018 Oct 8;28(11):1062–76. doi: 10.1038/s41422-018-0097-4 (PMC6218444; doi:10.1038/s41422-018-0097-4)
Supplement: Supplementary file 9 — Supplementary information, Figure S9 [file 41422_2018_97_MOESM9_ESM.pdf]

Figure S9

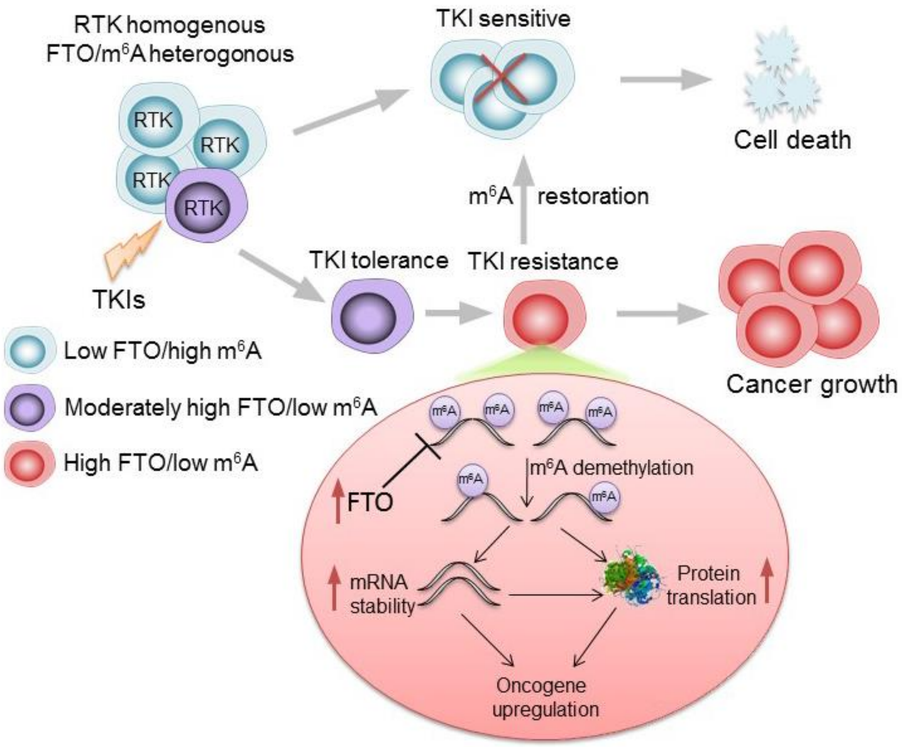

Figure S9. Schematic model illustrating the role of the FTO-m^{6}A axis in mediating the development of TKI resistant phenotypes. Distinct m^{6}A abundance exists in naïve cancer cells that are genetically homogeneous; TKI exposure induces m^{6}A demethylation resulting from FTO upregulation; m^{6}A depletion confers resistance to TKIs by upregulating m^{6}A-bearing survival genes; Reversal of m^{6}A marks in resistant cells restores sensitivity to TKIs.
