# Supplementary material for: A dynamic N6-methyladenosine methylome regulates intrinsic and acquired resistance to tyrosine kinase inhibitors
Source: Cell Res. 2018 Oct 8;28(11):1062–76. doi: 10.1038/s41422-018-0097-4 (PMC6218444; doi:10.1038/s41422-018-0097-4)
Supplement: Supplementary file 12 — Supplementary information, Table S3 [file 41422_2018_97_MOESM12_ESM.pdf]

Table S3. Clinical characterizations of AML patients receiving nilotinib therapy

**Patients**	**n = 14**
Age, median	58 (24-65)
Male gender	76.5%
Hemoglobin, gm/dL	9.3 (7-14.5)
WBC, $\times 10^{9}$/L	7 (0.5-124.7)
Platelets, $\times 10^{9}$/L	52 (18-85)
Bone marrow blasts	68% (21-97)
Peripheral blood blasts	30 (0-82)
Normal cytogenetics	41%
FLT3 mutated	4/15 (27%)
